# Suggested visual blockade during hypnosis: Top-down modulation of stimulus processing in a visual oddball task

**DOI:** 10.1371/journal.pone.0257380

**Published:** 2021-09-15

**Authors:** Marcel Franz, Barbara Schmidt, Holger Hecht, Ewald Naumann, Wolfgang H. R. Miltner

**Affiliations:** 1 Institute of Psychology, Friedrich Schiller University of Jena, Jena, Germany; 2 Institute of Psychology, University of Trier, Trier, Germany; La Sapienza University of Rome, ITALY

## Abstract

Several theories of hypnosis assume that responses to hypnotic suggestions are implemented through top-down modulations via a frontoparietal network that is involved in monitoring and cognitive control. The current study addressed this issue re-analyzing previously published event-related-potentials (ERP) (N1, P2, and P3b amplitudes) and combined it with source reconstruction and connectivity analysis methods. ERP data were obtained from participants engaged in a visual oddball paradigm composed of target, standard, and distractor stimuli during a hypnosis (HYP) and a control (CON) condition. In both conditions, participants were asked to count the rare targets presented on a video screen. During HYP participants received suggestions that a wooden board in front of their eyes would obstruct their view of the screen. The results showed that participants’ counting accuracy was significantly impaired during HYP compared to CON. ERP components in the N1 and P2 window revealed no amplitude differences between CON and HYP at sensor-level. In contrast, P3b amplitudes in response to target stimuli were significantly reduced during HYP compared to CON. Source analysis of the P3b amplitudes in response to targets indicated that HYP was associated with reduced source activities in occipital and parietal brain areas related to stimulus categorization and attention. We further explored how these brain sources interacted by computing time-frequency effective connectivity between electrodes that best represented frontal, parietal, and occipital sources. This analysis revealed reduced directed information flow from parietal attentional to frontal executive sources during processing of target stimuli. These results provide preliminary evidence that hypnotic suggestions of a visual blockade are associated with a disruption of the coupling within the frontoparietal network implicated in top-down control.

## Introduction

Multiple studies have demonstrated that hypnotic suggestions can significantly influence the cognitive and emotional processing of sensory stimuli while persons are hypnotized. The term “hypnotic suggestion” denotes here all types of verbal recommendations addressed by a hypnotist to someone else about what this person might perceive, feel, think, memorize, or do while hypnotized. For example, well hypnotized individuals commonly report significant changes of the gestalt of objects [1–3], modulations of colour [4–6], hue [4] and other physical properties of visual stimuli [7]. Further studies have reported deafness or modulated loudness [8], and changes of the smell of olfactory stimuli [9]. Others demonstrated reduced or no pain in response to real noxious events, when suggested that a virtual anaesthetic substance (cream, fluids) would block somatosensory receptors of their skin [10–16].

All of these studies have shown that hypnotic suggestions might have strong power over how people perceive the world. So far, however, there is only limited knowledge about the specific cognitive mechanisms and the roles of different brain structures involved in these hypnotically induced changes [17–20]. Several theories of hypnosis have more or less explicitly linked hypnotic phenomena to suggestion-induced modulations of high-level cognitive functions such as executive monitoring and/or cognitive control [21–26] that guide and control bottom-up processes in lower-level brain regions [27]. It is now widely accepted that top-down modulation of sensory processing is mediated by long-range signals originating from prefrontal and parietal brain regions [28], also referred to as executive control network [29] or frontoparietal network. This network includes the dorsolateral prefrontal cortex as a major hub and parietal regions in the inferior parietal lobule/sulcus, and the precuneus [30]. In general, this network is believed to be instrumental in tasks that require focused attention, working memory, and response selection [29].

Functional neuroimaging research has provided evidence in support of the notion that hypnotic phenomena are implemented by top-down regulation via a frontoparietal network. For example, one study found that suggested paralysis was characterized by a profound reconfiguration of activity within prefrontal and parietal areas of both hemispheres, which was distinct from simulation of paralysis and from voluntary inhibition of motor responses in the normal state [31]. Another neuroimaging study investigated the effects of hypnosis during a Stroop task and provided evidence of atypical prefrontal functioning involving regions implicated in conflict monitoring and control processes [32]. During visual colour hallucination, highly suggestible participants revealed less activity in the primary visual cortex and greater activation in parietal and frontal areas relative to low suggestible participants when they were suggested to see shades of grey while looking at colour images [33]. Further electrophysiological research revealed that highly suggestible individuals exhibited lower frontoparietal phase synchrony in the upper alpha frequency band (10.5–12 Hz) during hypnosis than low suggestibles suggesting that high hypnotic suggestibility may be linked to a functional disruption of the frontoparietal network [34]. These results not only imply that hypnotic effects and its underlying mechanisms may be closely linked to the frontoparietal control network, but also specifically affect brain areas related to the suggested task.

The purpose of the present study is to investigate whether hypnotic suggestions of a visual blockade would influence the connectivity profile of the executive control network, thereby modulating the information processing in posterior attentional and lower-level brain areas of visual processing. To address this question, we extended the analysis of data already published in an earlier paper of our group [35] in which even-related potentials (ERP) were used to investigate behavioural and neural effects of hypnotic suggestions of a visual blockade (HYP) on the processing of visual stimuli compared to a control condition (CON) where no suggestions were given. In both conditions, participants were requested to count rare target stimuli while ignoring the frequent and rare stimuli. The results showed that hypnotic suggestions led to a significant reduction in the P3b ERP amplitude, which was strongly associated with impaired counting performance. While the first paper focused on ERP amplitude effects at two electrode sites (Fz, Pz), the present study investigated the influence of hypnotic suggestions on: a) the topographical distribution of attention- and task-related ERP components (i.e., the N1, P2 and P3b amplitudes), b) their underlying neural source dynamics that account for the activity of the frontal executive control system and sources in posterior brain areas involved in the control of attention and stimulus-task associations, and c) the interactive connectivity profiles between these sources by means of effective connectivity.

## Materials and methods

### Participants and data

Sixty healthy participants (30 women and 30 men, mean age 23.1 years, age range of 18–44 years) took part in the study after giving written informed consent. Participants were recruited at the Friedrich Schiller University and assigned to three equally-sized subgroups according to participants’ suggestibility level (low, medium, high) as revealed by the Harvard Group Scale of Hypnotic Suggestibility. In HYP, participants received several suggestions to enter hypnotic trance followed by suggestions that a virtual wooden board in front of their eyes would obstruct the perception of the screen (see S2 File). While imagining the obstruction participants completed a visual oddball task (see below). In CON, participants completed a similar visual oddball task but without being hypnotized and without suggestions of obstruction. Each participant completed the HYP and CON condition in one experimental session, with the order of the two conditions counterbalanced across participants. On average, participants received a financial bonus of € 24 for participation (€ 6 per hour). The study protocol was approved by the ethics review board of the School of Social and Behavioural Sciences of the University of Jena and was in line with the declaration of Helsinki.

### Stimuli and procedure

A visual three-stimulus-oddball paradigm was employed in which participants were presented with a random sequence of three coloured geometric stimuli (squares/circles/triangles), termed as targets, standards, and distractors, respectively. These stimuli were either (blue/yellow/red) or (green/ brown/violet) with the order of the two colour sets being pseudorandom across conditions. Stimuli were presented with probabilities of 0.1, 0.8, and 0.1, respectively. Participant’s task was to silently count the targets. The oddball task consisted of approximately 540 (±10) stimuli and lasted about 13.5 minutes. Stimuli were presented at the center of a video monitor which was approximately 95 cm in front of participants while they sat on a comfortable chair in a shielded and dimly lit EEG chamber. During stimulus presentation, participant’s eye movements were supervised with an eye-tracking device. The geometric stimuli subtended horizontal and vertical visual angles of approximately 4.6° x 4.6°. Each stimulus appeared for 500 ms and consecutive stimuli were separated by a random inter-stimulus interval of 1 to 2 s. The experiment was programmed and presented by means of Presentation^®^ software (Version 17.1, Neurobehavioral Systems, Inc., Berkeley, CA, www.neurobs.com). In addition, a second experimental section followed each oddball task both in HYP and CON conditions where visual steady-state stimuli were presented. However, this experimental section is not subject to this study and therefore not covered here. Furthermore, participants received a German short version of the Inventory Scale of Hypnotic Depth (ISHD) at the end of the hypnosis condition. This version included 20 items assessing several common experiences individuals report when hypnotized (maximum score: 20).

### EEG recording and pre-processing

Brain electrical activity was recorded from 64 sintered Ag/AgCl electrodes (EASYCAP GmBH, Herrsching-Breitbrunn, Germany) that were placed on the participants’ heads according to the 10–20 system. Remaining electrodes were interspaced equally between these 21 sites. During recording, all channels were referenced online to the FCz electrode. The impedances of all electrodes were kept below 10 kΩ. EEG and EOG signals were registered using two BrainAmp amplifiers and the BrainVision Recorder software (both Brain Products, Gilching, Germany). Following analogue band-pass filtering (0.05–500 Hz), continuous EEG signals were digitized with a sampling rate of 1 kHz and stored to hard disk for later offline analysis. EEG data were preprocessed using EEGLAB ([36], Version 13.6.5b). Datasets were down-sampled to 250 Hz and re-referenced to average reference for further processing. The following analysis steps were chosen in view of the subsequent source analysis and therefore differ slightly from our previous paper [35]. The datasets were pruned from artifacts related to eye-blinks and ocular movements using independent component analysis (ICA). Therefore, a duplicate of the re-referenced EEG dataset was offline band-pass filtered (*pop_eegfiltnew*) from 1−40 Hz with a transition bandwidth of 1 Hz (highpass) and 10 Hz (lowpass), respectively, using a Hamming windowed sinc finite impulse response (FIR) band-pass filter and subsequently segmented into continuous 1 s intervals. The dataset was then pruned from unique, nonstereotyped artifacts by applying higher order statistic functions (*pop_jointprob*, *pop_rejkurt*) to electrode channels in order to discard data segments containing unlikely EEG values (>3 SD) that are indicative of artifacts [37]. Extended infomax ICA, as implemented in EEGLAB, was then applied to the pruned dataset.

The original re-referenced EEG dataset was filtered offline using a Hamming windowed sinc FIR band-pass filter from 0.1–40 Hz with a transition bandwidth of 0.1 Hz (highpass) and 10 Hz (lowpass), respectively. Subsequently, the ICA demixing matrix was applied to this dataset. Components representing eye-blinks or ocular movements were identified using the fully automated method EyeCatch [38] and subtracted from data [39]. The dataset was segmented into epochs from −0.2 to 1.0 s relative to stimulus onset and baseline corrected using the pre-stimulus interval from −0.2 to 0 s. The epochs were pruned from non-stereotyped artifacts (*pop_jointprob*, *pop_rejkurt*) by discarding epochs with amplitude values >3 SD. On average, 43.2% (Min: 22.3%, Max: 60.1%) of all trials were retained after artifact rejection. In hypnosis/control condition, the mean number of trials amounted to ≈24/≈22 valid target trials (*Min*: 11/12, *Max*: 38/33), ≈24/≈22 distractor trials (*Min*: 8/13, *Max*: 33/31), and ≈197/≈188 standard trials (*Min*: 83/122, *Max*: 259/282) for each participant. ERP waveforms were averaged separately for each participant, stimulus type and experimental condition HYP and CON. These preprocessed datasets were imported into SPM12 (v7219; http://www.fil.ion.ucl.ac.uk/spm) for EEG sensor analyses and subsequent EEG source reconstruction [40].

The EEG analysis focused on the investigation of event-related potentials in response to the three visual stimulus types and scrutinized three so-called late potentials, i.e., the N1, P2, and P3b components: The N1 component in response to visual stimuli is a large, negative-going late ERP component that commonly peaks between 150 to 200 milliseconds post-stimulus. It is present all over the scalp with a common maximum of its amplitude over fronto-central brain areas of both hemispheres. When the stimulus is presented laterally, its maximum emerges on the contralateral hemisphere, in case of central application it displays bilaterally. When a stimulus is presented centrally, the N1 is bilateral. The N1 component is especially relevant as a neural signature of early attentional processes and gets maximal when attentions selectively allocated to a predefined target stimulus and its physical features. Furthermore, it was shown repeatedly that its amplitude is not affected by hypnosis [10, 11, 13, 14]. In regard to the present study, we expect the same magnitude and source configuration of the N1 component under hypnotic suggestion of a visual blockade as in the control condition described below.

The visual P2 amplitude is a positive going electrical potential that peaks between about 150 and 275 ms with a common maximum at around 200 ms post stimulus onset. Its topographic maximum is more fronto-central with a maximum around the vertex but like the N1 also to observe all over the scalp with different magnitudes. Its amplitude gets affected by stimulus features such as colour, size, and orientation of the stimulus and by task features like attention, repetition and probability of the stimulus. Some studies have additionally shown that the magnitude of the P2 varies positively with the intensity and brightness of a stimulus whereas pure hue seems not to be significant [41, 42].

The positive-going P3b represents a prominent brain-wide activity that was heavily investigated in the past. According to [43, 44], the P3b component reflects cognitive processes associated with the analysis of stimulus probability, the recognition, and categorization of stimuli. Verleger [45] recently emphasized, that the P3b amplitude is an important signature for the association of a stimulus to task relevancy. All of these cognitive processes include attentional processes and comparison to and updating of internal representations of stimuli, i.e., long-term and especially working memory functions. According to the triarchic concept of the P3b, suggested by Johnson [46], the magnitude of P3b amplitude was shown to vary as a function of stimulus distinctiveness and participant’s attention (E), stimulus probability P, and meaning or task relevancy (M) of a stimulus according to the following formula: [P3b_amp_ = f(E x (P + M))]. Investigations of the neural generators of the visual P3b convert to a few matching brain areas that have potential relevance in the constitution of the P3b. Brain damage studies and intracranial recordings have suggested the temporoparietal junction (TPJ) [47, 48], modality-specific brain regions of the sensory cortex, multimodal association areas along the superior temporal gyrus (STG), the posterior parietal cortex (PPC), and the medial temporal lobe and hippocampus as potential sources of the P3b [49–52]. Further studies using ERP and fMRI methods have identified bilateral activities in the medial and ventral prefrontal cortex (PFC), the supra- (SPL) and inferior parietal lobe (IPL), the cingulate Gyrus (CG) [53]. A fMRI-constrained source analysis of Bledowski et al. [54], which is especially interesting for the present study due its application of a similar paradigm, identified P3b sources in parietal, inferior, ventrotemporal brain areas, and in higher visual and supramodal association areas. These sources have also been confirmed by studies using Loreta (see [55–60]). All these observations suggest that a complex network of frontal, temporal and parietal brain areas is involved in the generation of the P3b.

### Distributed source reconstruction

For EEG source reconstruction, we employed the parametric empirical Bayesian (PEB) framework and the multiple sparse priors (MSP) method as implemented in SPM. SPM’s template head model was used for computing the forward model of each participant’s brain. The head model comprised four meshes based on the cortex, inner skull, outer skull, and scalp. The distributed model approach constrained the source space to the vertices of the cortical surface mesh. Default 3D locations, being hard-coded in SPM, were assigned to EEG sensors in order to link between the coordinate system of EEG sensors and the coordinate system of the template structural MRI image. A lead field matrix (forward solution) was then computed using a three-shell Boundary Element Model (inner skull, outer skull, and scalp meshes). Prior to source estimation, spatial and temporal data reductions were conducted to increase the signal-to-noise ratio (SNR). Subsequently, datasets were subjected to group-based source reconstruction using the MSP-approach as inversion type and Greedy Search as the fitting algorithm. The time window of inversion ranged from −100 to 600 ms. The results of source estimation were averaged over the P3b window (320−470 ms), and exported to MNI space as surface-based GIFTI images. For each participant, a set of 2-by-3 (condition, Stimulus-Type) GIFTI images based on the P3b window was then created and used for statistical analysis (see below). See S1 File for further details on source reconstruction.

### Statistics

#### Sensor-level analysis

The sensor-level analysis was conducted separately for the N1 (80−168 ms), P2 (168–272 ms), and P3b (320−470 ms) window. For each participant, a set of 2-by-3 (condition, stimulus type) NIFTI images (3D sensor-space x time volumes) was created for the respective latency window. For group statistical analysis of the 3-by-2-by-3 design (suggestibility-group, condition, stimulus-type), we opted for the partitioned error approach (random effects analysis). The NIFTI images were first transformed into a set of differential effects for each participant, i.e., first level contrast images (within-subject analysis) were created using the ImCalc facility in SPM. This resulted in four sets of first level contrast images to test for three main effects, three 2-way interaction effects and one 3-way interaction effect. For each set of contrast images, two General Linear Models (GLM) were specified (using one-/two-sample *t*-test designs or 1-way ANOVA designs) in SPM, and estimated at the second level, and relevant *t*- or *F*-contrasts were specified to test for the effects of interest. The significant interaction effect was followed up by focused contrasts (simple effects) by specifying a 2-by-3 (condition, stimulus-type) repeated measures ANOVA within the GLM framework of SPM. To improve sensitivity of contrasts, we added 60 participant columns into the GLM that serve to remove between-subject variance. For further details on GLM specification and testing see S1 File. Effect sizes for within-subject ANOVA designs, expressed as partial eta squared $\eta_{p}^{2}$, were calculated from the *t*-contrast (*t*-value) and its degrees of freedom using the formula [61]:

$$\eta_{p}^{2}=\frac{F*df_{effect}}{F*df_{effect}+df_{error}}\;\text{where}\;F(1,\text{df})=t-\text{value}.$$

#### Source-level analysis

Based on the results in sensor space, the source-level analysis was restricted to the P3b window, and the significant interaction effect (condition, stimulus-type) in sensor space was followed up by focused contrasts at the source-level. In line with the sensor analysis, we specified a 2-by-3 (condition, stimulus type) repeated measures ANOVA within the GLM framework using the GIFTI images of the distributed source reconstruction. All SPM results at sensor- and source-level are reported with family-wise-error (FWE) corrected *p*-values at the cluster level (*p* < .05, cluster-level FWE correction, cluster forming threshold *p* < .001 uncorrected). GLMs were estimated using Restricted Maximum Likelihood estimation. For labeling of anatomic structures obtained by source reconstruction, we employed the command-line tool *atlasquery* supplied with FSL (FMRIB Software Library, v6.0, Oxford, UK) by interrogating the Harvard-Oxford cortical and subcortical structural atlases. Differences in latency of the peak of the source waveforms were analyzed using Wilcoxon signed-rank test due to non-normality of data. In addition, we tested whether poorer counting accuracy in HYP compared to CON correlates with changes in source activities within several brain regions by means of Pearson correlation. We employed QQ-plots to visually check whether data followed a normal distribution.

### Spectrotemporal connectivity analysis

Additionally, we tested whether the observed P3b effects of hypnosis at sensor- and source-level are associated with transient changes in effective connectivity (directed information flow) between different brain processes accompanying the oddball task. Several simulation studies indicate that multivariate effective connectivity measures are quite sensitive to data preprocessing transformations (e.g. filtering, downsampling) and can eventually lead to erroneous results such as missed or spurious interactions [62, 63]. Since reconstructed time series signals at source-level are based on several preprocessing steps (e.g. broadband filtering, spatial and temporal singular value decomposition) the connectivity analysis was applied to a subset of electrodes at sensor-level. Based on the source analysis, we selected a subset of three electrodes of the left hemisphere (O1, CP1, F5) that best represented sources located in occipital, parietal, and frontal brain regions. Corresponding electrodes of the right hemisphere (O2, CP2, F6) were included in a separate analysis. For this we employed single-trial data that were neither high- nor lowpass filtered in order to avoid possible influences of filtering on connectivity measures. Pairwise directed interactions between electrodes were derived from validated time-variant MultiVariate AutoRegressive (tvMVAR) models by computing the time-variant partial directed coherence (PDC) for a range of frequencies (1–30 Hz) within the segmented epochs (−1 to 1 s). The PDC is able to detect linear relationships among multivariate time series and was introduced to disentangle direct and indirect influences [64]. The Source Information Flow Toolbox (SIFT 1.4.1; [65]) was used for model fitting, validation, and computation of the spectrotemporal connectivity metric. For further details on data preprocessing, modeling, and the applied connectivity metric see S1 File.

A nonparametric cluster-based permutation approach was employed to test for significant conditional differences (hypnosis vs. control) within the spectrotemporal connectivity matrices of the pairwise channel combinations. The clustering was performed over adjacent time-frequency bins and samples whose *t-*value surpassed the cluster-defining threshold (*α*-level = .05) were assigned to clusters. Cluster-level statistics were calculated by taking the sum of *t-*values within each cluster. The empirical cluster-level statistics were then compared against the reference distribution of the maximum/minimum cluster-level statistics (_max_Σ, two-sided *t-*test) that was approximated by Monte Carlo simulations (*n* = 1000 permutations). Cluster-based statistics were computed for the time-frequency window ranging from 200 to 600 ms post-stimulus and from 1 to 20 Hz. The time-frequency window was chosen to contain the main activity window of the P3b. The restriction to the lower frequency range was motivated by suggestions of Buzsaki et al. [66] that communication between distant neural sources preferentially happens in lower frequencies while closely neighboring networks mainly communicate in the frequency range of gamma. Clusters were considered significant at *α*-level = .025 (two-sided). The statistical significance of each cluster was corrected for multiple comparisons in the time- and frequency domain using the cluster-based correction method as implemented in FieldTrip (version: 20191113; http://fieldtriptoolbox.org; [67]).

## Results

The results section is organized around three central issues outlined in the Introduction: (1) the section on ‘Sensor analysis’ extends the Schmidt et al. [35] study by additional topographic analyses of the N1, P2, and P3b ERP components. (2) Based on the results of the sensor analysis, the subsequent ‘Source analysis’ investigated P3b effects related to hypnosis at source-level. (3) Finally, we examined whether effects of hypnosis are linked to modulations of the interactive connectivity profile between frontal, parietal, and occipital electrodes by studying the effective connectivity (partial directed coherence).

### Behavioural data

**Fig 1:**
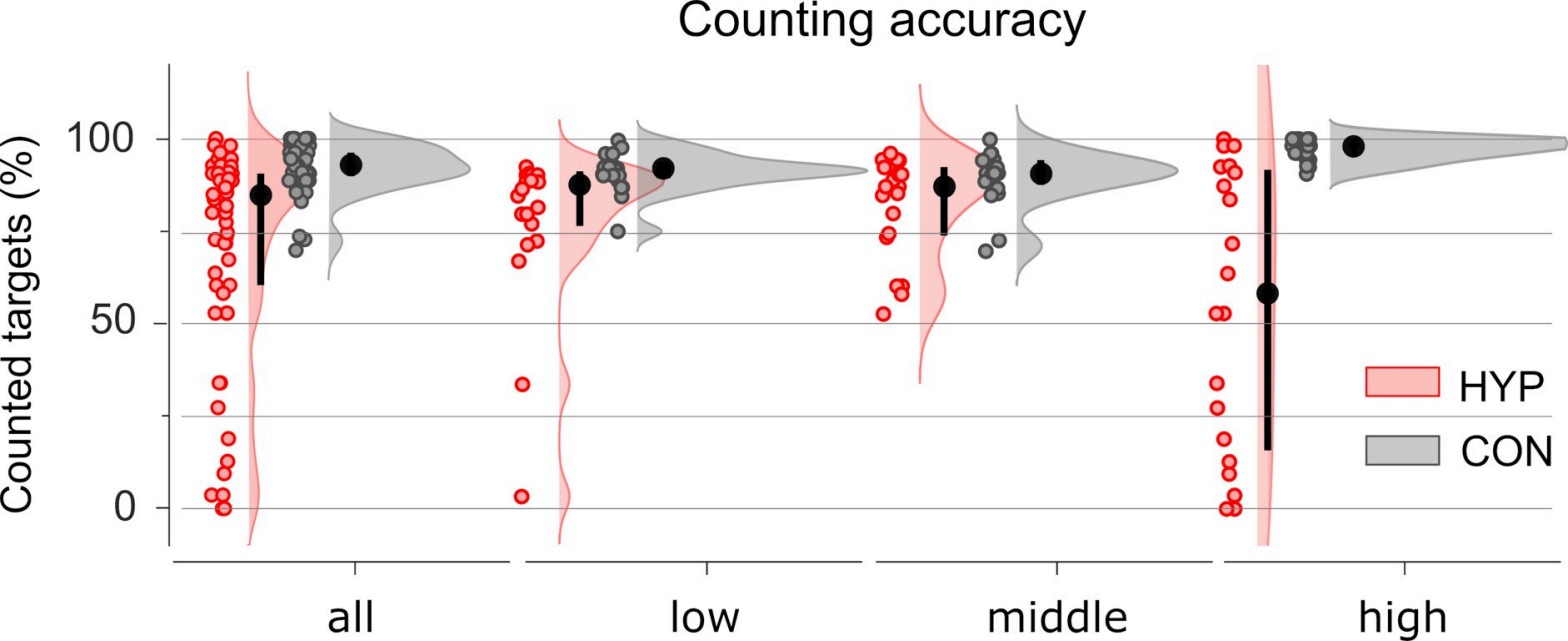
Counting accuracy in hypnosis (HYP, red) and control (CON, grey) depicted separately for the total group of participants (all) and the three subgroups of low, middle, and highly suggestible participants. Counting accuracy (%) was defined as the number of silently counted targets relative to the total number of targets presented. Black circles and lines mark the median and the interquartile range (difference between 75th and 25th percentiles), respectively. Pink and grey areas indicate the probability density function of the corresponding data points.

Since the analysis of behavioural data was already presented in detail in the previous paper [35], only the main behavioural results of this study are covered here. Participants’ self-reported depth of hypnosis as assessed by a short version of the ISHD (20 items) was on average 9.1 (Min: 4, Max: 15). During CON, participants counted an average of 93% of the target stimuli correctly (Fig 1), while under HYP participants’ counting accuracy was about 20% worse than under CON. With an average of 43% of target stimuli failing, counting accuracy was particularly impaired in highly suggestible participants, and even some participants did not count any target stimuli at all in HYP. Furthermore, highly suggestible participants counted more accurately during the CON condition than middle and low suggestible participants, whereas the latter do not differ from each other.

### Sensor analysis

#### N1/P2 component

Maximum N1 amplitudes of target, distractor, and standard stimuli in HYP and CON were observed on average 115 ms post-stimulus (108–132 ms) at frontocentral electrodes (Fz, FCz, AFz). The P2 peak amplitudes were observed on average 231 ms post-stimulus (212–252 ms) at parietal electrodes (POz, PO4) for all stimuli types in HYP and CON except for the target in HYP with maximum amplitudes at FCz.

For N1 and P2 amplitudes, only the main effect of Stimulus-Type reached significance (S1-1 and S1-2 Figs, S1-3 and S1-4 Tables in S1 File). Importantly, there were no significant differences in amplitude between HYP and CON for both components.

#### P3b component

Maximum P3b amplitudes of target stimuli in HYP and CON were observed at Pz at 408 ms post-stimulus, whereas maximum P3b amplitudes of distractor and standard stimuli occurred at 320 ms post-stimulus at electrode PO4. In accordance with Johnson’s triarchic model of P3b amplitude [46], target stimuli were largest since they fulfilled two critical parameters of the model: i.e., they were task relevant and infrequent stimuli. Next largest P3b amplitudes were observed for distractor stimuli. They only fulfilled one of the critical parameters, i.e. stimulus probability. They were presented rarely but had no task-relevancy. Finally, the smallest amplitudes were obtained for standard stimuli; they were presented frequently and had also no task-relevancy.

**Fig 2:**
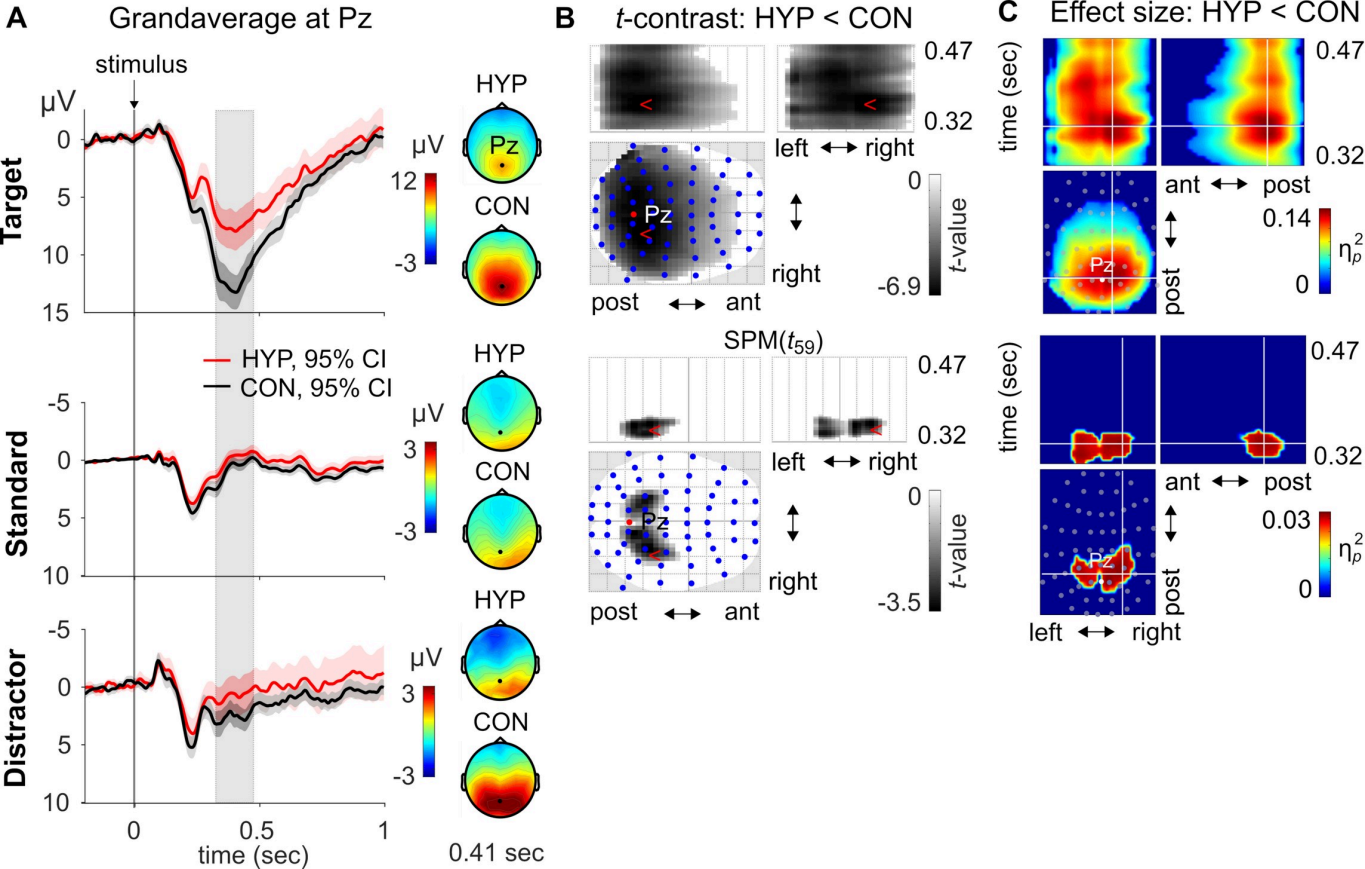
**(A)** Grandaverage waveforms (pooled across all participants, *n* = 60) and 95% C.I. in hypnosis (HYP, red) and control condition (CON, blue) for target, distractor, and standard stimulus type at electrode Pz. The grey rectangle marks the P3b time window (320–470 ms) used for statistical analysis of data at sensor- and source-level. Sensor-level topographies at the peak latency of the target P3b (410 ms) depending on condition and stimulus type. **(B)** For each stimulus type a one-sample *t*-Test was performed and the results were assessed with a *t*-contrast to test for differences between experimental conditions, i.e., HYP < CON, within the P3b time window as shown in (A). There were no significant amplitude differences for the distractor between HYP and CON. The summary statistic scalp-time images were thresholded at *p* < .001 (uncorrected) with FWE correction at cluster-level, *p* < .05, based on random field theory. The statistical parametric maps (SPMs) are displayed as Maximum Intensity Projection (MIP) of the 3D (scalp x time) summary statistic image. Blue dots mark the electrode sites. post = posterior; ant = anterior **(C)** Effect size, partial eta square ($\eta_{p}^{2}$), SPMs display the temporal evolution of the significant *t*-Contrasts HYP < CON for the target and standard shown in (B). Grey dots mark the electrode sites and the white lines’ crossing the peak effect size of the space x space or space x time map.

The three-factorial analysis of P3b amplitudes within the window of 320 to 470 ms revealed a significant main effect for Stimulus-Type (S1-5 Table in S1 File); P3b amplitudes were largest for target stimuli, followed by distractor stimuli, and smallest for standard stimuli (S1-3 Fig in S1 File) replicating the common oddball effect. Additionally, we observed a significant main effect for Condition (S1-4 Fig, S1-6 Table in S1 File). There was no significant main effect for Suggestibility-Group. Likewise, the two-way interaction of Condition by Suggestibility-Group and Stimulus-Type by Suggestibility-Group, and the three-way interaction of Condition by Stimulus-Type by Suggestibility-Group were also not significant. Most importantly, we found a significant two-way interaction of Condition by Stimulus-type (S1-5 Fig, S1-7 Table in S1 File) indicating that P3b amplitude differences between HYP and CON varied by Stimulus-Type. Since the factor Suggestibility-Group did not have any influence on the P3b amplitudes, data were collapsed across this factor in subsequent analysis including source and connectivity analyses. Fig 2A displays the grandaverage P3b waveforms at electrode Pz for the target, distractor, and standard stimuli in HYP and CON (see also S1-5A Fig in S1 File) and the corresponding topographies at the peak latency 410 ms of the target P3b (Fig 2A). To disentangle the Condition by Stimulus-type interaction, a simple effects analysis was conducted. The corresponding scalp-time SPMs for the *t*-Contrast of HYP < CON separated by Stimulus-Type are shown in Fig 2B. The P3b amplitudes of the target were significantly smaller in HYP vs. CON (S1-8 Table in S1 File); the effect was most pronounced at right parietal electrode sites (Cohen’s *d =* −0.89; Fig 1C) peaking at 372 ms post-stimulus. However, this P3b difference between both experimental conditions was not significantly correlated with the hypnotic experience as examined by the ISHD. Furthermore, amplitudes within the P3b window of the standard stimulus were significantly smaller in HYP vs. CON (Fig 2B and S1-8 Table in S1 File) with maximal effect size of Cohen’s *d =* −0.45 at left parietal electrodes at 344 ms. The P3b of the distractor stimuli did not significantly differ between HYP and CON.

### Source analysis–P3b

**Fig 3:**
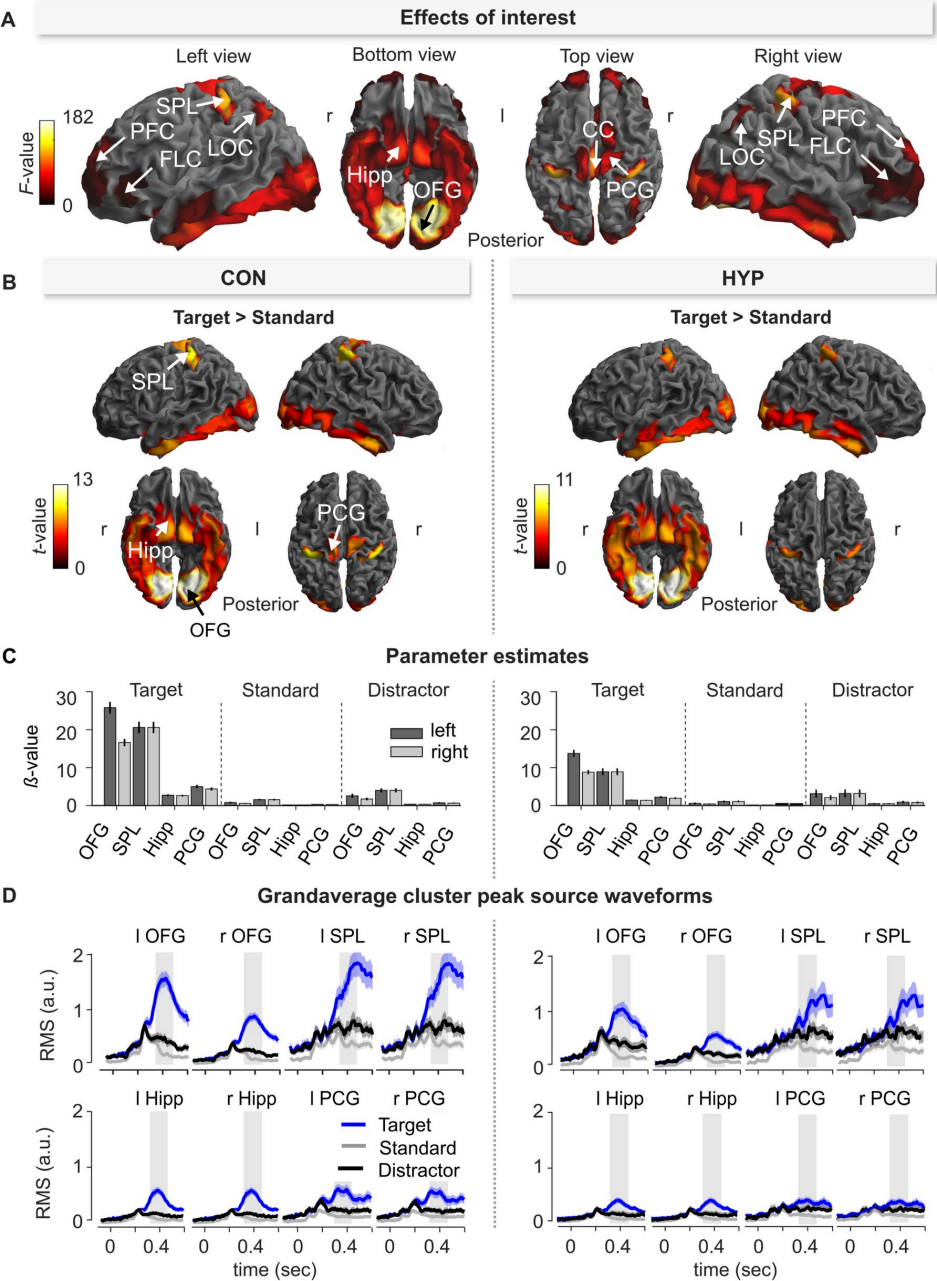
Source-level effects of the three-stimulus-oddball paradigm during the P3b window (320–470 ms) in the control (CON) and hypnosis (HYP) condition. **(A)** Effects of interest (*F*-contrast) showing sources whose activation was significantly different from zero. r = right; l = left. **(B)** Statistical comparison (*t*-contrast) of target > standard for source activities within the P3b window in the CON and HYP. The cortical images show sources that were significantly more activated following processing of target as compared to standard stimulus. The labeled brain structures refer to the cluster peaks. PFC = prefrontal cortex; FLC = frontolateral cortex; SPL = superior parietal lobule; LOC = lateral occipital cortex; Hipp = Hippocampus; OFG = occipital fusiform gyrus; CC = posterior cingulate cortex; PCG = precentral gyrus. The summary statistic images of the cortical mesh were thresholded at uncorrected *p* = .001 with FWE correction at cluster-level, *p* = .05, based on random field theory. **(C)** Parameter estimates (*β*-value, 90% C.I.) of target, distractor and standard in CON and HYP for sources located within cluster peaks of the left and right hemisphere (see Fig 3B and S1-10 Table in S1 File). **(D)** Grandaverage cluster peak source waveforms and 95% C.I. Since polarity of source waveforms is meaningless, and depends, inter alia, on the orientation of the cortical surface where the sources in question are located, values of source strength are expressed as root mean square (RMS, in arbitrary units). Grey rectangle marks the P3b window.

This section addresses the question of which cortical sources contributed to the target P3b effects described in the previous section. Fig 3A illustrates sources whose activations within the P3b time window were significantly different from zero (*F*-contrast: effects of interests). Accordingly, a large-scale network of spatially-distributed regions is involved in the processing of visual stimuli during the P3b window including the ventral visual stream, lateroccipital, parietal, posterior cingular, and frontal brain areas (see S1-9 Table in S1 File for statistics).

#### Target vs. standard

To specifically examine target related activity of the P3b at source-level, we contrasted the processing of target stimuli against standard stimuli in the CON and HYP condition. The resulting ‘target network’ for CON (left) and HYP (right) is depicted in Fig 3B, showing larger source activities for target as compared to standard stimuli. Target processing constituted a similar large-scale network of multiple sources both in HYP and CON. The *t-*contrast (target > standard) in HYP and CON resulted in four pairs of bilateral significant source clusters that are statistically summarized in S1-10 Table in S1 File. The four bilateral clusters in CON were located in the ventral visual stream with cluster peaks in the occipital fusiform gyrus (OFG) and in the dorsal visual stream with cluster peaks in the superior parietal lobule (SPL). Furthermore, there were significant source activities in the hippocampal area (Hipp) and precentral gyrus (PCG). The parameter estimates (90% C.I.) at corresponding cluster peaks and grandaverage waveforms of source activity (root mean square, RMS) are displayed for each stimulus type in Fig 3C and 3D for HYP and CON, respectively. All clusters show larger source activities for target stimuli than for standard and distractor stimuli indicating that these sources mainly generated the P3b scalp potential. During target processing, the source activities in left and right SPL revealed the broadest and largest activities peaking at 438 ms and extending beyond 600 ms (Fig 3D). The second largest source amplitudes were observed in the OFG of the left and right ventral visual stream peaking at 390 ms, and hence earlier than SPL. In contrast, the bilateral source clusters in Hipp and PCG peaking at 375 ms and 363 ms, respectively, were clearly less pronounced than those of SPL and OFG, and hence, contributed less to the P3b component.

#### HYP vs CON

**Fig 4:**
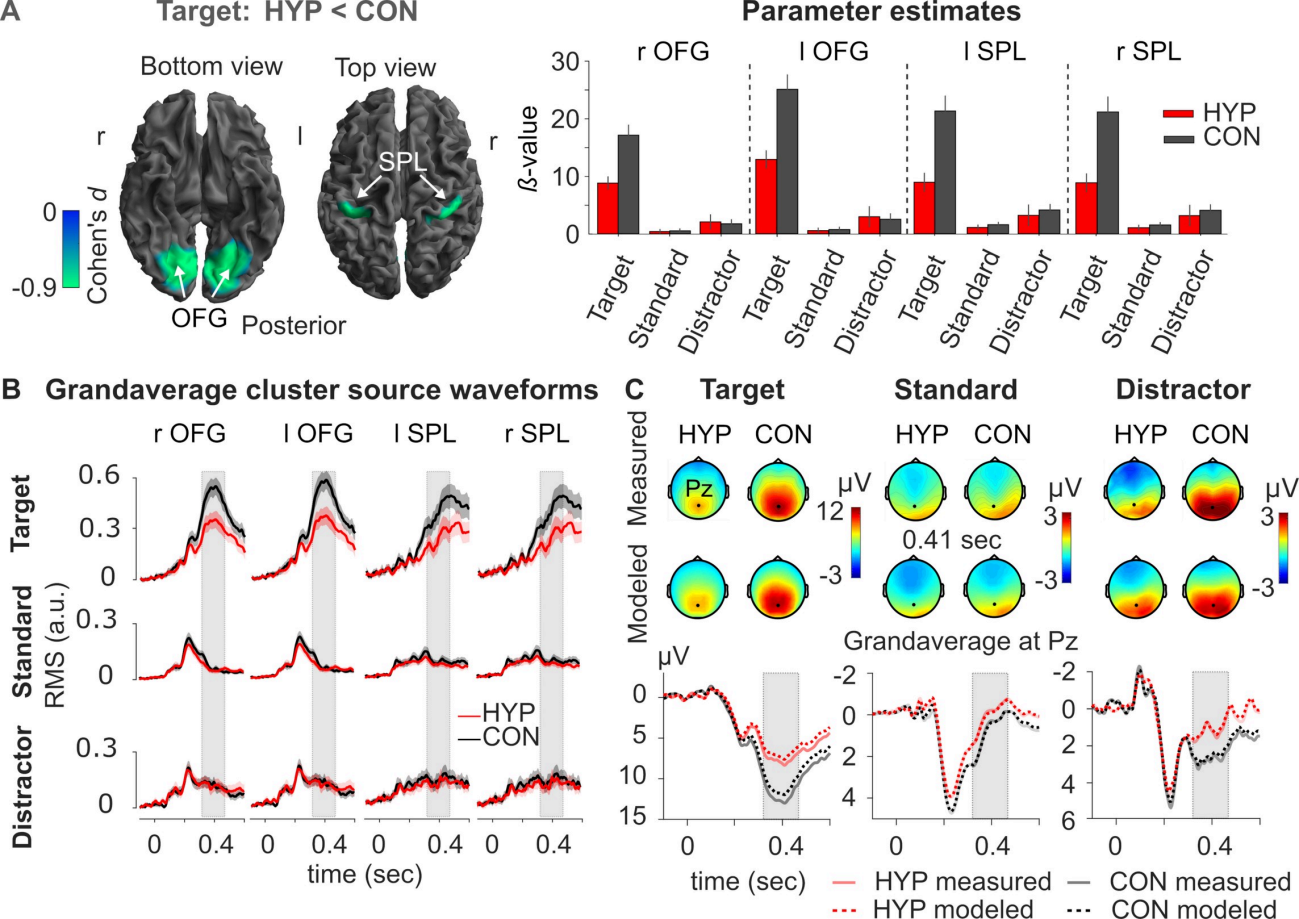
Effect of hypnosis at source-level. **(A)** Effect size ($\eta_{p}^{2}$) of statistical comparison (*t*-contrast) of hypnosis (HYP) vs. control (CON) for the processing of the target stimulus. Left panel: The cortical mesh images show sources that were significantly less activated in HYP compared to CON following processing of target. SPL = superior parietal lobule; OFG = occipital fusiform gyrus. The summary statistic images of the cortical mesh were thresholded at uncorrected *p* = .001 with FWE correction at cluster-level, *p* = .05, based on random field theory. r = right, l = left. Right panel: The bar plots summarize the contrast estimates (90% C.I.) for three stimulus types in HYP and CON at the respective peaks of the cluster. **(B)** The grandaverage cluster source waveforms (across participants, *n* = 60) and 95% C.I. are based on the average of source waveforms within the respective clusters found in Fig 3A (see S1-11 Table in S1 File) for the three stimulus types in HYP (red) and CON (black). Source strength is expressed as root mean square (RMS, in arbitrary units). Grey rectangle marks the P3b window (320–470 ms). **(C)** Comparison between modeled (dotted lines) grandaverage data, i.e., all back-projected sources, and measured (solid line) grandaverage data in sensor space at electrode Pz for three stimulus types in HYP (red/pink) and CON (black/grey). Note that back-projected model data at sensor-level (modeled data) match very well the measured data profiles as indicated by grandaverages at Pz and the scalp topographies at 0.41 s.

Since target processing was significantly reduced during HYP compared to CON at sensor-level, we examined where these P3b differences were reflected at the source-level. During HYP, target processing was associated with significantly less activation in the OFG and SPL of both hemispheres compared to CON (target: HYP < CON; S1-11 Table in S1 File). Fig 4A depicts the respective source statistics and the corresponding parameter estimates (90% C.I.) at the peaks of the significant clusters. The grandaverage source waveforms of the respective cluster peaks are illustrated in Fig 4B. SPL source activities peaked significantly later than OFG source activities, both in HYP (*Mdn*_OFG_ = 388 ms vs. *Mdn*_SPL_ = 466 ms, *z* = −2.79, *p* = .005, *r* = −0.25) and CON (*Mdn*_OFG_ = 394 ms vs. *Mdn*_SPL_ = 448 ms, *z* = −3.74, *p* = .002, *r* = −0.34), as shown in Fig 3B. Importantly, the comparison of peak latencies between HYP and CON for OFG and SPL sources yielded no significant differences. Unsurprisingly, given the results of the sensor analysis, the source analysis revealed no brain region that exhibited enhanced source activities during HYP. Contrary to sensor-level results, we did not observe any significant difference of source activities between HYP and CON for standard stimuli.

In addition, we analyzed the correlation between counting accuracy and P3b source activities in several brain areas (for details see S1-12 Table in S1 File and S1-6 Fig in S1 File). The P3b source activities at OFG (*r* = −0.49, *p* = < .001) and SPL (*r* = −0.27, *p* = .03) were significantly associated with counting accuracy. The lower the source activations in the OFG and SPL under HYP compared to CON, the worse was the counting accuracy.

The quality of source reconstruction can be seen in Fig 4C. In general, it shows that back-projected model data in sensor space (modeled data) correspond very well to the measured data profiles as indicated by grandaverages of target, standard, and distractor stimuli at Pz and the corresponding scalp topographies at 0.41 s.

### Spectrotemporal connectivity analysis

Finally, we tested whether the observed P3b effects of hypnosis at sensor- and source-level were associated with transient changes in effective connectivity (directed information flow) between an occipital (O1), parietal (CP1), and frontal electrode (F5) of the left hemisphere by means of the Partial Directed Coherence (PDC) method. Results of the multivariate autoregressive model estimation and validation can be found in the S1 File (S1-7 Fig in S1 File).

**Fig 5:**
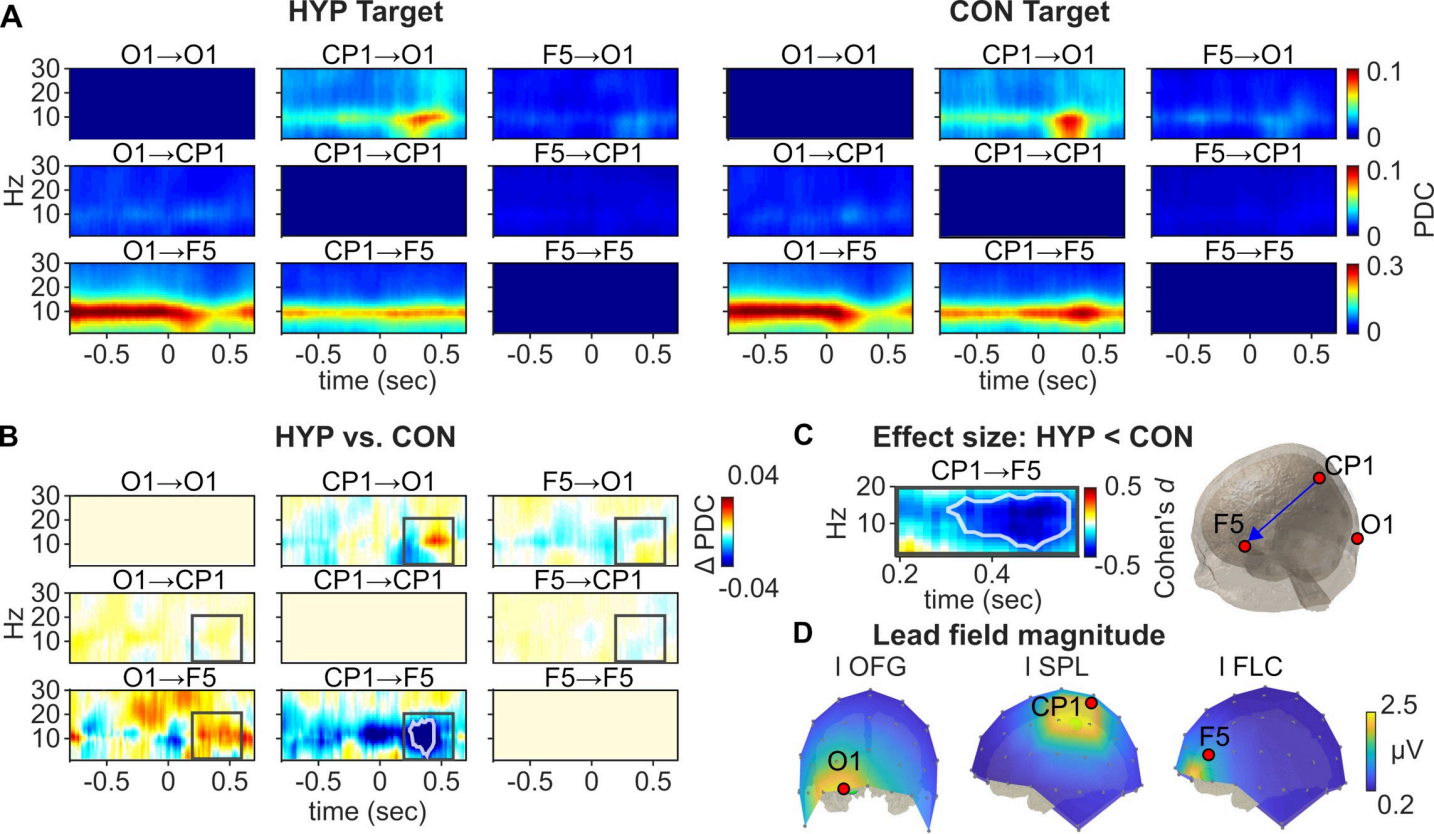
Spectrotemporal connectivity within the network of three electrodes (O1, CP1, F5) at the left hemisphere across all participants (*n* = 60). **(A)** Evolution of the pairwise connectivity during the hypnosis (HYP, left) and control (CON, right) condition for the target stimulus. The connectivity values represent the time-variant partial directed coherence (PDC) from 1 to 30 Hz within the latency range of −800 to 700 ms relative to the target stimulus. Note, that directed information flow is mainly located in the low frequency range (8−12 Hz) both in HYP and CON. **(B)** Absolute difference of the connectivity matrices between HYP and CON. Warmer colours (red) indicate HYP > CON and cooler colours (blue) CON > HYP. The grey rectangle marks the spectrotemporal region of interest (ROI) that was used for statistical comparison of both conditions. **(C)** Effect size expressed as Cohen’s *d* for the statistically significant ROI difference in spectrotemporal connectivity between HYP vs. CON. White lines in (B) and (C) encircle the significant spectrotemporal region at *p* < .05 corrected for multiple comparisons in the time-frequency plane using cluster-based statistics. **(D)** Lead field magnitude (μV) of three source cluster peaks located in the left hemisphere: the occipital fusiform gyrus (l OFG), the superior parietal lobe (l SPL), and the frontolateral cortex (l FLC). The maximum lead field magnitude of these source cluster peaks defined three electrodes as suitable representatives of these structures that were selected for the connectivity analysis.

Fig 5A depicts the grandaverage spectrotemporal connectivity matrix between three electrodes of the left hemisphere (O1, CP1, F5) in HYP (left panel) and CON (right panel) for target stimuli across all participants. Across all channel pairs, the left occipital electrode O1 exhibited the strongest amount of causal outflow within the analyzed network (Fig 5A). From electrode O1, the information flows to the right frontal electrode F5 over a constant frequency band (6−14 Hz) during the period prior to the presentation of the target stimulus (−800 to 0 ms) with connectivity gradually subsiding at post-stimulus time. This connectivity pattern is both present in the HYP and CON condition. A similar connectivity pattern can be observed between the right centroparietal electrode CP1 and the electrode F5. Here, the outflow of information from CP1 to F5 is limited to a narrow frequency band (9–11 Hz) during pre- and post-stimulus time in HYP and CON, although the connectivity becomes stronger during the post-stimulus time in case of the CON condition. The electrode CP1 exhibits another connectivity to the O1 site which is limited to the low frequency range (5−10 Hz) and most prominent during the post-stimulus time of the P3 component both in HYP and CON.

The absolute difference between the connectivity matrices for HYP vs. CON is shown in Fig 5B for the target stimulus. Since CON was subtracted from HYP, warmer regions (red) indicate stronger connectivity values during HYP, and cooler regions (blue) indicate stronger connectivity during CON. The grey rectangle marks the spectrotemporal region of interest (ROI) that was used for statistical comparison of both conditions. Crucially, the centroparietal electrode CP1 exhibited a significantly reduced information flow to the frontal electrode F5 within the ROI (1–20 Hz; 200–600ms) during HYP in comparison to the CON condition (cluster statistic Σ_*t*_ = −479.61, *p* = .024) while this effect was mainly located in the alpha frequency range (8−12 Hz). The effect size (Cohen’s *d*) of the statistically significant ROI cluster is shown in Fig 5C. No further significant conditional differences were found. Additionally, these results were substantiated by a further connectivity analysis with corresponding electrodes of the right hemisphere (O2, CP2, F6) that yielded a very similar pattern of results (S1-8 Fig in S1 File). Fig 5D illustrates the lead field magnitude of the three cluster peaks located in the left occipital (l OFG), left parietal (l SPL) and left frontal (l IFG) regions. The lead field topographies of these cluster peaks were used to define the three electrodes (O1, CP1, F5) in sensor space as suitable representatives of these regions.

## Discussion

In the present experiment, participants completed a visual oddball paradigm composed of target, standard, and distractor stimuli during a hypnosis (HYP) and a control (CON) condition. During HYP, participants were suggested that a virtual wooden board in front of their eyes would obstruct their view of the video screen. In each condition, they were asked to count the rare visual target stimuli presented on the screen. Based on the assumptions of several theories of hypnosis, as mentioned in the introduction, i.e., that hypnotic suggestions are implemented by modulating top-down processes originating from frontal and parietal brain regions, the purpose of the present study was to investigate the influence of the suggested visual blockade on visual stimulus processing by examining its effects on a) the topographical distribution of ERP components (i.e., the N1, P2, and P3b amplitudes), b) their underlying neural source dynamics, and c) the interactive connectivity profiles between frontal, parietal and occipital sources.

### Behavioural data

During HYP, participants committed significantly more counting errors than during CON. This effect was most pronounced among highly suggestible participants. The present study thus reveals that the hypnotically suggested virtual wooden board could significantly impair the counting performance associated with perception and categorization of target stimuli. We argue that highly suggestible participants generally complied better with task instructions, and showed better counting accuracy in CON but were significantly hampered by the hypnotic suggestion when counting during HYP. This hypnosis-related impairment of visual perception confirms earlier studies, e.g., on visual gestalts [1–3], colour [4–6], and hue [4].

### Sensor analysis of the N1, P2 and P3b ERP components and their topographical distribution

#### N1/P2 component

Latency and topography of the N1-component corroborates earlier investigations [41, 68] and reveals that N1 reflects basic sensory processing, i.e., the perception of and differentiation between the three visual stimuli, the allocation of early attentional resources to these stimuli, and suppression of attention to other stimuli in the experimental room. Data reveal that these processes were not affected by hypnosis. Topography and latency of P2-component also replicates observations of earlier studies [41, 68]. Its functional meaning is associated with feature-based attention, identification and characterization of visual objects. P2 amplitude did not show significant differences between the HYP and CON and also no sign. effects of suggestibility. These equivalent early brain responses to visual stimuli in both conditions indicate that participants attended the stimuli equally well. Participants thus were not blinded by the suggestion or so much distracted that the observed behavioural effects during HYP would solely reflect blurred visual acuity, defocusing, or completely looking away from the screen.

#### P3b component

The topographical analysis nicely reflects the well-known distribution of the P3b component, i.e., the association of stimulus probability and task relevancy with P3b amplitude (for review see [46, 69]). Target P3b amplitudes were significantly affected by the hypnotic suggestion: they were smaller and topographically more lateralized in the right posterior hemisphere and more spatially focused as compared to CON. Something similar was observed for the standards, but here differences between HYP and CON were very small. In contrast, P3b amplitudes to rare distractor stimuli were not affected significantly during HYP as compared to CON and their topography was also temporooccipital like for the standards but less focused than for targets. This is an interesting observation, since the lack of a significant difference of P3b amplitude between HYP and CON indicates that the suggestion during HYP, the wooden board would obscure the perception of *all stimuli*, did not affect the task-irrelevant to-be-ignored distractor, but only the task-relevant to-be-counted/attended target stimuli. It seems that the paradox of suggestion, on the one hand not to perceive the stimuli, but on the other hand to count the targets, was solved by participants by switching the attentional resources on and off and maximizing attention to the targets, at least once in a while, in order to comply with the suggested counting task, i.e., mapping the targets to counting. This change in attention to targets could explain that about 73% of the target stimuli were counted correctly on average across all participants under HYP. According to the functional properties of the P3b amplitude as a signature of attention, stimulus probability, meaning, and assigning a stimulus to a specific task [45], it is therefore not surprising that the P3b amplitudes in response to targets during HYP are smaller than during CON. Obviously, the hypnotic state and the suggestion of the visual blockade do not suppress the perceptual processing of the visual stimuli, but induce an unstable state of attention that allows correct counting from time to time.

Notably, the effects of HYP in terms of P3b amplitudes did not significantly differ between low, medium, and high suggestible participants. This finding deviates from our earlier study [35] in which we observed a significant interaction between experimental conditions and suggestibility. We assume that these divergent results are likely due to different statistical methods used in both papers. While the P3b amplitude analysis of the former study was restricted to one electrode only (Pz), the analysis of the current study included scalp-time data of all 64 electrodes and their corresponding amplitude values within the P3b window (320–470 ms). Although the current approach uses random field theory–which is more sensitive than a Bonferroni correction for the number of tests–to control for the implicit multiple comparison problem inherent to EEG data (due to the large number of time bins and sensors, [70]), the current inference may be less sensitive than the single electrode analysis in [35]. In other words, the effects of suggestibility at sensor-level could be rather small overall and spatially very narrowly circumscribed, so that the method used in this case was not sensitive enough to detect these effects.

### Source analysis–P3b

The present source analysis of the P3b amplitudes is based on a relatively new method of ERP source reconstruction using a parametric empirical Bayesian (PEB) framework and a multiple sparse priors (MSP) method as implemented in SPM (for details see S1 File).

Compared to earlier P3b source reconstructions under normal non-hypnotic conditions of visual information processing using the Brain Electrical Source Analysis method [71] or Loreta [59, 60, 72], our source analysis corroborated very similar sources identified in [54] using a similar three-stimulus-oddball paradigm. For the processing of all three stimulus types in both conditions, symmetrical sources were identified in ventrotemporal (OFG), occipital (LOC), parietal (SPL), frontal (PFC, FLC), precentral (PCG), and hippocampal (Hipp) structures of both hemispheres and two midcentral sources in the posterior cingulate cortex (PCC), cf. Fig 3A. All these sources mirror well-known brain areas that comprise the cortical processing system of visual stimuli. With regard to the processing of target stimuli in HYP and CON, P3b was especially constituted by the OFG, SPL, Hipp and PCG sources.

The source analysis yields supportive evidence for a differential activity and involvement of visual neural sources in the processing of targets between HYP and CON and underlines the significant importance of the neural generators of P3b in OFG and SPL. They seem to be indispensable for focused attention in order to identify and classify the stimuli correctly and relate them to defined tasks. Furthermore, there is clear evidence that the activity of both sources is nicely associated with the counting accuracy of targets. The lower the activation of the OFG and SPL sources under HYP compared to CON, the worse was the counting accuracy. The OFG as part of the ventral visual-processing stream or “what system” is supposed to perform fine-grained analysis of visual stimuli, including processing of shape and colour [73], and appears to be concerned with classifying the stimuli as task-relevant (counting) or non-task-relevant. Regarding SPL, various neuroimaging studies have provided evidence for its involvement in top-down (goal-directed) attentional orienting (for reviews see: [74, 75]) and when attention is focused to target stimuli [76]. Thus top-down attentional signals from SPL could bias the processing in low-level visual areas towards task-relevant stimuli, with attention-related effects having been reported to cross multiple visual areas from V2 through V4 within the ventral visual stream [76]. There is also evidence that SPL is involved in linking relevant sensory information to actions for a given task [74, 77–79], also referred to as stimulus-response mapping. Accordingly, one parsimonious view would suggest that regions in the parietal cortex (SPL) may bias the information processing in lower visual areas (OFG) and additionally relay the presence of a target to frontal executive monitoring/control structures, which then initiate counting in left frontal areas [80–82]. The tasks of OFG and SPL further require some differences in timing. Stimulus classification should precede the forwarding of target presence to the frontal brain areas. This is exactly what we observed for both sources (see Figs 3D and 4B), i.e., maximal activity of the OFG precedes the activity of SPL. The fact that source activity was stronger when targets were correctly identified and counted, thus supports the role of SPL in attention and mapping target stimuli to counting. In contrast, when counting was impaired/missed during HYP, source activity was significantly reduced in both source clusters as compared to CON. Like at the sensor-level, the obstructive hypnotic suggestions of the visual board resulted in a reduction of source activity of the right and left OFG by over 50% which was not observed for the distractor and standard stimuli. Activities of OFG and SPL sources hardly differed while processing the latter two stimuli between HYP and CON. Analysis of the latency of maximum source activities also revealed no significant differences between HYP and CON suggesting that cognitive processes organized by these brain sources are not generally delayed during HYP. Although the source reconstruction method employed here already uses an advanced template head model, it nonetheless contains some inaccuracies in the modeling of each participant’s head. Its localizations are only approximate and therefore, the labeling of sources by means of the used atlases is only tentative.

### Spectrotemporal connectivity analysis

The connectivity analysis of P3b-related frequency bands (i.e., 1−30 Hz) was realized for three pairs of electrodes at either the left (O1, CP1, F5) or right hemispheres (O2, CP2, F6). These electrodes were identified as best representatives of the cluster sources OFG (electrode O1/O2) and SPL (CP1/CP2) of the left and right hemispheres, and according to the effects of interest analysis–depicted in Fig 2 –as a source of the executive system of the left and right prefrontal area (F5/F6). Results of the connectivity analyses showed that both frontal and parietal electrodes expressed significant differences in directed connectivity within the alpha frequencies of P3b activities between HYP and CON. Less strong connectivity between these electrodes was observed during HYP as compared to CON. For all other frequency fractions of the P3b activity between 1 and 30 Hz there was no further significant difference of pairwise channel combinations between HYP and CON. This reduced information flow was not identified so far during hypnosis, but in relation to the sensor- and source-level data of the P3b amplitude it could indicate that the parietal source informs the lateral-prefrontal area whether the respective stimulus should be counted and stored in subnetworks that organize arithmetical functions [83, 84].

We assume that this information is critically involved in the generation of counting errors during the visual obstruction suggestion while participants are hypnotized. If this holds true across further studies, this suggestion associated modulation of information flow from parietal brain areas to frontal brain areas might support the role of the frontoparietal network for executive monitoring and control during hypnotic suggestion of a visual blockade. That this information flow is restricted to a narrow frequency band additionally supports observations that information exchange between neural networks across larger distances commonly happens at low frequencies, whereas closely neighbored neural networks preferentially communicate using faster frequencies [66, 85].

One weakness of our study relates to the fact that the source analysis model used, in contrast to other concepts such as Loreta or BESA, usually identified very large, widespread sources, especially in the parietal structures that include the entire so-called ventral path of visual processing. For this reason, we are not able to differentiate detailed sources for the analysis of the three visual stimuli, e.g. whether our visual blockage suggestions had specific effects on the colour or the pattern of the three stimuli and whether the poor identification of the stimuli under hypnosis was differentially effective compared to the other experimental manipulations in subdivisions of the visual path. This remains reserved for further studies and, above all, for a comparative application of the Loreta or BESA approaches with this new source model.

A further caveat of the present study might relate to the way how we assed participants’ behavioral responses to target/nontarget and distractor stimuli during each experimental condition. Since participant were requested to count the targets and report them at the end of the experimental conditions this response measure only represents an integrated memory-based measure and no online measure taken after each single target presentation. In focusing on targets only, we also don’t know exactly how the other two stimuli were attended and perceived. In planning this experiment, we discussed this problem long and intensively, however refrained from a single trial assessment due to the potential danger that the hypnotic trance of participants might have been disrupted, and the danger that attention allocation to the three types of stimuli would have become blurred. With reference to numerous oddball-studies in which targets were also counted in silence and in which clear differences in ERP amplitudes were observed in response to targets, non-targets, and distractor stimuli (for review see [46]), we opted for our memory-based approach. Nevertheless, studies with a trial-based counting approach would represent interesting additions to the current study.

### Summary

Taken together, the P3b amplitude turns out to be an informative parameter for the investigation of cognitive mechanisms of hypnotic suggestions and supports at least for visual events earlier assumptions by Crawford [86] that the modulation of attention and task execution represents important mechanisms of hypnotic action. The HYP condition is characterized by a reduced directed flow of information between parietal and frontal brain areas that have been linked to the executive control network. If this parietofrontal interaction is functionally modulated by hypnotic suggestions, the proper recognition and categorization of stimuli and their assignment to certain stimulus-related tasks (e.g. counting) can be impaired. This result partly supports several theories postulating that hypnosis is implemented by top-down modulation of stimulus processing.

## Supporting information

S1 File Additional methods and results. (PDF)

S2 File Wording of experimental instructions and tests. (PDF)
