# Supplementary material for: Suggested visual blockade during hypnosis: Top-down modulation of stimulus processing in a visual oddball task
Source: PLoS One. 2021 Sep 15;16(9):e0257380. doi: 10.1371/journal.pone.0257380 (PMC8443036; doi:10.1371/journal.pone.0257380)
Supplement: S1 File — (PDF) [file pone.0257380.s001.pdf]

Supplemental information I to the article:

Suggested visual blockade during hypnosis:
Top-down modulation of stimulus processing in a visual oddball task

Marcel Franz[1*], Barbara Schmidt[1], Holger Hecht[1], Ewald Naumann[2] and Wolfgang H.R. Miltner[1]

[1] Institute of Psychology, Friedrich Schiller University of Jena, Jena, Germany

[2] Institute of Psychology, University of Trier, Trier, Germany

Materials and Methods

Bayesian distributed source reconstruction

In the following section, we briefly present the parametric empirical Bayesian (PEB) framework and the multiple sparse priors (MSP) method as implemented in SPM to invert electromagnetic models and to localize distributed sources of MEG/EEG datasets (for details see: 1, 2). In contrast to equivalent current dipole models (3, 4) which assume a small number of current dipoles (i.e., active sources), the distributed model approach uses a large set of dipoles – fixed in position and orientation – that are distributed over the cortical surface mesh (i.e., search or source space). The distributed source model can be represented as a multivariate linear model involving a linear mapping between dipole moments of a large number of distributed current dipoles and a set of MEG/EEG measurements recorded by sensors placed on the head (5) according to:

$$Y = LJ + \varepsilon \quad (1)$$

where $Y \in \Re^{N_c \, x \, N_t}$ is the dataset formed by N_c channels and N_t time samples, $L \in \Re^{N_c \, x \, N_d}$ is a known leadfield or gain matrix (forward model) linking dipole moments (i.e. amplitude of the dipolar currents sources) of N_d sources to the scalp EEG channels in Y, $J \in \Re^{N_d \, x \, N_t}$ is the matrix of unknown source currents (parameters), and ε is a zero-mean Gaussian random noise term.

Since the problem of model inversion is ill-posed, prior information (empirical priors) must be included to find a unique solution to the MEG/EEG source reconstruction problem. From a Bayesian viewpoint, a prior is a probability density that defines the uncertainty of an unknown parameter before the data is taken into account (6). In recent years, the PEB framework has been applied to the source reconstruction of MEG/EEG data (7, 8) that imposes flexible constraints on the inverse solution.

Parametric empirical Bayes (PEB) approach

The source reconstruction model as expressed in Eq. (1) can also be formulated in the context of a two-level hierarchical empirical Bayes model under parametric assumptions, i.e., additive Gaussian random effects at each level (8):

$$Y = LJ + \varepsilon_1 \quad (2)$$

$$J = \varepsilon_2 \quad (3)$$

where both ε_1 and ε_2 are sampled from a Gaussian distribution with zero-mean: $\varepsilon_1 \propto N(0, Q_\varepsilon)$ and $\varepsilon_2 \propto N(0, Q_J)$. Q_ε and Q_J are spatial covariance matrices in sensor and source space, respectively. Both matrices are expressed as a weighted linear combination of various independent covariance components C_i (8) :

$$Q_\varepsilon = \lambda_1^{(1)} C_1^{(1)} + \lambda_2^{(1)} C_2^{(1)} + \cdots \quad (4)$$

$$Q_J = \lambda_1^{(2)} C_1^{(2)} + \lambda_2^{(2)} C_2^{(2)} + \cdots \quad (5)$$

where $\lambda_i^{(l)}$ is the unknown "hyperparameter" for the i-th covariance component of the l-th level (7). The first-level hyperparameters $\lambda^{(1)}$weight the covariance components of sensor noise and the second-level hyperparameters $\lambda^{(2)}$ weight the contribution of multiple empirical covariance priors on the sources (1). Due to its hierarchical form, the parameters of the second (source) level impose an empirical prior on the level below (sensor).

In the PEB framework of SPM, the hyperparameters are iteratively adjusted using a variational Bayesian estimation scheme by maximizing an objective or cost-function in terms of the variational "free-energy" which provides an approximation to the model evidence (9). It follows that the source estimates, we seek to compute, can be expressed as the expected value of the source currents conditioned upon the data Y (2): $\hat{J} = E[p(J|Y)]$. This estimate is given by (5):

$$\hat{J} = Q_J L^T \left(Q_\varepsilon + L Q_J L^T\right)^{-1} Y. \quad (6)$$

Since the data Y are known, and the lead-field matrix can be calculated based on a physical head model, the main objective is to find good estimates of Q_ε, and Q_J in order to calculate the source currents $\hat{J}$ (2). A sensor noise covariance matrix Q_ε could be based on empiric evidence (empty room recordings) or in the absence of prior information, one could assume a matrix with a single covariance component $Q_\varepsilon = \lambda_1^{(1)} I_{N_c}$, where $I_{N_c} \in \Re^{N_c \, x \, N_c}$ is an identity matrix with ones one the main diagonal and zeros elsewhere, and $\lambda_1^{(1)}$ is the sensor noise variance (2).

Numerous constraints can be employed as priors on the covariance matrix of sources Q_J. The selected set of prior components defines the prior assumptions of the model. A classical source reconstruction approach such as the Minimum Norm solution (MMN), for example, assumes that all sources are uncorrelated and have similar prior variances (minimum norm prior): here, the prior covariance matrix of the sources takes the form of an identity matrix $Q_J = I_{N_d}$.

Multiple sparse prior

The currently implemented multiple sparse priors (MSP) algorithm (1) of SPM considers the prior source covariance matrix Q_J as a weighted sum of N_p prior components, also referred to as Empirical Bayes:

$$Q_J = \sum_{i=1}^{N_p} \left(\lambda_i^{(2)}\right) C_i^{(2)}. \quad (7)$$

where each $C_i^{(2)} \epsilon \, \Re^{N_d \, x \, N_d}$ is a prior source covariance matrix (2). For the sake of convenience, we consider the case where each prior component represents a single potentially activated cortical patch. Here, the hyperparameters $\lambda^{(2)} = \left\{\lambda_i^{(2)}, \ldots, \lambda_{N_p}^{(2)}\right\}$ weight these cortical patches (prior components) and

are pruned to those $C_i^{(2)}$ corresponding to activated regions, using the *free-energy* as a cost function (10). Note that these prior components can contain different classes of informative priors, e.g., anatomical and neurophysilogical knowledge, or fMRI priors (7). The currently implemented MSP algorithm of SPM constrains the source space to the vertices of the cortical surface mesh. Since the neural current flow has some local coherence, the original large set of dipoles ($N_d = 8196$ dipoles for the "normal" grid of SPM12) is reduced to a fixed set of locally smooth focal patches, e.g., SPM employs a set of $N_p = 512$ cortical patches (2). The centers of these patches are a sparse sample of the original set of dipoles – used to construct the lead-field matrix – and cover the entire cortical surface mesh.

EEG forward modeling

After converting the preprocessed single-subject datasets into a SPM compatible data format, SPM's template head model was used for computing the forward model of each subject. The template head model (volume conductor model) is based on the single-subject T1-weighted structural template image of the Montreal Neurological Institute (MNI). The head model comprised four meshes based on the cortex, inner skull, outer skull, and scalp. The cortical mesh of the template brain is a continuous triangular tessellation of the grey/white matter interface of the neocortex. A density of 8196 vertices (4098 per hemisphere) with mean inter-vertex distance of ≈5 mm was chosen for the template cortical mesh, and 2562 vertices for the template inner skull, outer skull and scalp meshes (7) corresponding to the "normal" grid selection in SPM. Default 3D locations, being hard-coded in SPM, were assigned to EEG sensors in order to link between the coordinate system of EEG sensors and the coordinate system of the template structural MRI image. A leadfield matrix (forward solution) was then computed using a three-shell Boundary Element Model (BEM) based upon inner skull, outer skull and scalp meshes. At each point (vertex) on the cortical mesh a lead field was computed that corresponds to the potential distribution on all sensors of a dipole oriented perpendicularly to the respective surface mesh point.

EEG inversion

Prior to source estimation, spatial and temporal data reductions were conducted to increase the signal-to-noise ratio (SNR) and to reduce the computational load of subsequent estimation (2). Therefore, sensors space was transformed to a subset of orthogonal sensors by applying singular value decomposition (SVD) over the outer product of the leadfield matrix with a cut-off of $> e^{-16}$ for singular values. This produced a set of m=58 orthogonal sensors (or spatial modes) for each subject. Similarly, using SVD with a cut-off $> e^{-8}$ for singular values, the temporal dimension reduction resulted in m = 6−16 temporal modes across subjects. Eventually, prepared datasets were subjected to group-based source reconstruction using the multiple sparse priors (MSP) approach as inversion type and Greedy Search (GS) as the fitting algorithm. The group-based inversion effectively restricts activity patches (source priors) to be the same across subjects while its activity can vary between them. The time window of inversion

ranged from −100 to 600ms and the frequency window of interest was set to default frequencies (0–256 Hz). The results of source estimation were averaged over the P3b window (320−470 ms) with the frequency window of interest set to default frequencies and contrast type to "evoked", and exported to MNI space as surface-based GIFTI images (values on a mesh). Images were finally smoothed by an isotropic 2D Gaussian kernel with 8 mm full width half maximum (FWHM) across the cortical mesh to better conform to assumptions of random field theory. For each subject a set of 2-by-3 (condition, stimulus type) GIFTI images based on the P3b window was created and used for statistical analysis (see below).

Statistics on sensor-level data

In the following section, we outline how the three factorial (Condition x Stimulus-Type x Suggestibility-Group) nature of our design was modeled and statistically tested in SPM12. We followed the partitioned error approach as outlined in the SPM wikibook (11). The subject-specific set of 2-by-3 GIFTI images (condition: c1 = hypnosis, c2 = control; stimulus type: s1 = target, s2 = standard, s3 = distractor) was arranged in the following order: c1s1 c1s2 c1s3 c2s1 c2s2 c2s3. This set was first transformed into four sets of contrast images using the spm_mesh_calc.m function and the respective contrast weights C of Table S1-1.

Table S1-1. Effects of interest and the corresponding contrast weights to calculate 1st level contrast images using the spm_mesh_calc function.

Effect of interest:	Contrast Image Set	1st level Contrast weights
E1 = ME: Group	1	$C_1 = [1\ 1\ 1\ 1\ 1\ 1]$
E2 = ME: Condition; E3 = INT: Group x Condition	2	$C_2 = [1\ 1\ 1\ \text{-1}\ \text{-1}\ \text{-1}]$
E4 = ME: Stimulus type; E5 = INT: Group x Stimulus-Type	3	$C_3 = \begin{bmatrix} 1 & \text{-1} & 0 & 1 & \text{-1} & 0 \\ 0 & 1 & \text{-1} & 0 & 1 & \text{-1} \end{bmatrix}$
E6 = INT: Condition x Stimulus type;<br>E7 = INT: Group x Condition x Stimulus type	4	$C_4 = \begin{bmatrix} 1 & \text{-1} & 0 & \text{-1} & 1 & 0 \\ 0 & 1 & \text{-1} & 0 & \text{-1} & 1 \end{bmatrix}$

E = effect, ME = main effect, INT = interaction

Then, for each set of contrast images, two General Linear Models (GLM) were specified and estimated to test for effects of interest. The respective factorial design specifications and 2nd level contrasts are summarized in Table S1-2.

Table S1-2. Factorial design specification.

**Effect of interest:**	**Contrast Image Set**[1]	**Factorial Design**	**2nd level Contrast Weights**
ME: Group	1	One-way ANOVA[a,c] (3 levels)	F-contrast $\begin{bmatrix} 1 & -1 & 0 \\ 0 & 1 & -1 \end{bmatrix}$
ME: Condition	2	One-sample t-test	F-contrast [1]
INT: Group x Condition	2	One-way ANOVA[a,c] (3 levels)	F-contrast $\begin{bmatrix} 1 & -1 & 0 \\ 0 & 1 & -1 \end{bmatrix}$
ME: Stimulus type	3	Two sample t-test[b,c]	F-contrast $\begin{bmatrix} 1 & 0 \\ 0 & 1 \end{bmatrix}$
INT: Group x Stimulus type	3	One-way ANOVA (6 levels, 2 for each group)[a,c]	F-contrast $\begin{bmatrix} 1 & 0 & -1 & 0 & 0 & 0 \\ 0 & 1 & 0 & -1 & 0 & 0 \\ 0 & 0 & 1 & 0 & -1 & 0 \\ 0 & 0 & 0 & 1 & 0 & -1 \end{bmatrix}$
INT: Condition x Stimulus type	4	Two-sample t-test[b,c]	F-contrast $\begin{bmatrix} 1 & 0 \\ 0 & 1 \end{bmatrix}$
INT: Group x Condition x Stimulus type	4	One-way ANOVA (6 levels, 2 for each group)[a,c]	F-contrast $\begin{bmatrix} 1 & 0 & -1 & 0 & 0 & 0 \\ 0 & 1 & 0 & -1 & 0 & 0 \\ 0 & 0 & 1 & 0 & -1 & 0 \\ 0 & 0 & 0 & 1 & 0 & -1 \end{bmatrix}$

[1] see Table S1-1
(a) independent measurements between levels (b) dependent measurements between levels (c) measurements in each level are assumed to have unequal variance

Connectivity analysis

The time series of the three electrodes were used to extract linear stochastic time-variant MultiVariate AutoRegressive (tvMVAR) models by fitting the ERP data from an ensemble of trials. The models were adaptively extracted over successive time windows using a short sliding window across the trial. This serves to promote local stationarity of the data within the window and yields time-resolved connectivity measures that are derived from these tvMVAR model parameters. To calculate spectro-temporal connectivity measures, a time-variant MVAR of the time series $\mathrm{X}(n) = [\mathrm{X}_1(n), \mathrm{X}_2(t), \dots, \mathrm{X}_d(n)]^T$ is estimated first, where $\mathrm{X}(n)$ represents the D-dimensional random process data vector of all signals/electrodes at time $\mathrm{n} = 1, \dots, N$, and N denotes the number of recorded data points. Let

$$\mathrm{X}(n) = \sum_{r=1}^{p} A_r(n) \times X(n-r) + E(n) \tag{8}$$

be a tvMVAR process of model order p which defines the number of included past time points with $X(n) \in \Re^D$, $A_r(n) \in \Re^{DxD}$ represents a matrix of AR coefficients for the r-th time lag, and $E(n) \in \Re^D$ a zero mean uncorrelated noise process.

Pairwise directed interactions between functional network nodes (electrodes) were derived from the validated tvMVAR models by computing the time-variant partial directed coherence (PDC). The PDC metric between the signals i and j of a multivariate system is defined as:

$$\mathrm{PDC}_{i \to j}(\omega, n) = \frac{\left|\tilde{A}_{ji}(\omega, n)\right|}{\sqrt{\sum_{k=1}^{D} \tilde{A}_i^*(\omega, n) \times \tilde{A}_i(\omega, n)}} \tag{9}$$

where $\tilde{A}_{ji}(\omega, n)$ represents the Fourier transformed time-variant AR coefficients, i.e. the causal influence from $i \to j$ at frequency ω, and $\tilde{A}_i$ denotes all outflows from i, and $\tilde{A}_i^*$ the complex conjugate. Hence, the PDC represents the outflow from $i \to j$, normalized by the total outflows from i and indicates the influence from $i \to j$ when the influence due to all other time series of the analysis is discounted.

The Source Information Flow Toolbox (SIFT 1.4.1; https://sccn.ucsd.edu/wiki/SIFT; (12)) was used for model fitting, validation and computation of the spectro-temporal connectivity metric. In order to mitigate possible influences of filtering on connectivity measures, single-trial data of the target stimulus were specially preprocessed for the purpose of the connectivity analysis. Specifically, continuous data were down-sampled to 500 Hz, referenced to average reference, 50 Hz notch filtered (FieldTrip-Toolbox function: ft_preproc_dftfilter), ocular artifact corrected using ICA, segmented into epochs from −1.0 to 1.0 s, and pruned from non-stereotyped artifacts (*pop_jointprob*). No high- or lowpass filter was applied. The mean number of target trials in hypnosis/control condition amounted to ≈39/≈37 (Min: 23/20, Max: 49/53). For each subject, we randomly sampled EEG trials (without replacement) from the condition with the larger number of valid trials to match the smaller number of trials for the other condition. For each subject and condition, single-trial ERP time series were detrended and normalized

across time and the ensemble of trials to remove the nonstationarity in the mean and standard deviation of the ERP (13). The MVAR models were fitted for each subject and condition separately, by applying the Vieira-Morf algorithm to the preprocessed time series signals using a sliding window (length: 300 ms, step size: 20 ms). The optimal model order was estimated by the Hanning-Quinn information criterion (HQIC) by automatically determining the minimum of the HQIC curve that was computed for a range of possible model orders between 1 and 30. For each subject, the model order p was separately determined for the hypnosis and control condition and the larger of both conditions was selected to match the two conditions. Subsequently, model validation criteria (whiteness-residuals test, percent consistency, and stability index) were computed for each tvMVAR model. The autocorrelation function (ACF) was used to test the whiteness of model residuals and thus to assess the model's ability to capture all temporal dynamics inherent to the time series signals. The percent consistency was computed for each sliding window within the epoch to evaluate the amount of concordance between the correlation structure of the real data and that of the modeled data. The stability index was employed for each window of the epoch to assess whether the model represents a stable process, i.e. the random process is stationary. Finally, the spectro-temporal connectivity estimator (PDC) was computed from the validated tvMVAR model for a range of frequencies (1–30 Hz) within the segmented epochs (−1 to 1 s). The results of model validation are summarized in the result section of this document.

RESULTS

SENSOR-LEVEL ANALYSIS

- **N1 window (80–168 ms)**

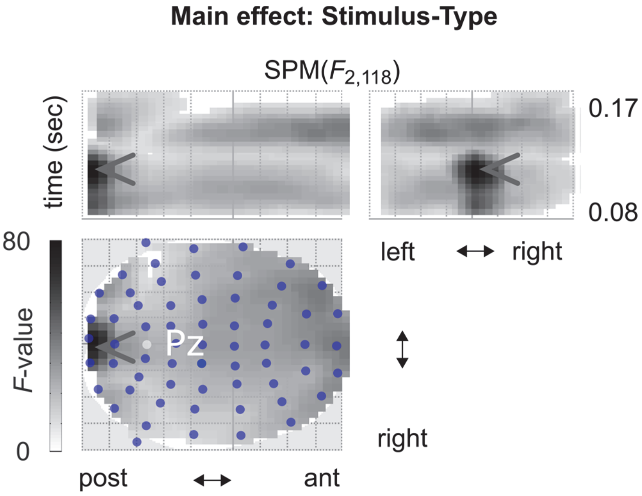

Figure S1-1. Main effect of Stimulus-Type within N1 window (80−168 ms). A paired-sample *t*-test (within-subject) was performed and the results were assessed with F-contrast. The summary statistic scalp-time data volume was thresholded at $p < .001$ (uncorrected) with FWE correction at cluster-level, $p < .05$, based on random field theory. Blue dots mark electrode sites and the grey arrows point to the global maximum. For details, see **Table S1-3**.

Table S1-3. Main effect of factor Stimulus-Type within N1 window (80−168 ms). Statistical results of scalp-time analysis.

Cluster-level		Peak-level				
$P_{FWE\text{-}corr}$	K_E	*F*-value	$P_{uncorrected}$	mm	mm	ms
<.001	13035	80.25	<.001	0	−59	112

Statistics: $p_{FWE\text{-}corr}$ = Family-wise-error corrected *p*-values adjusted for search volume. K_E = cluster with *K* elements. Height threshold: $F = 7.33$, $p = .001$ (uncorrected); df = (2, 118); Extent threshold: $k = 0$ bins; Smoothness FWHM = 65.6 88.9+ 51.0 mm mm ms; Expected bins per cluster <*k*> = 135.4; Search vol.: 1756502 = 19223 bins = 13.8 resels; Bin size: 4.2 5.4 4.0 mm mm ms; (resel = 1186 bins).

- **P2 window (168–272 ms)**

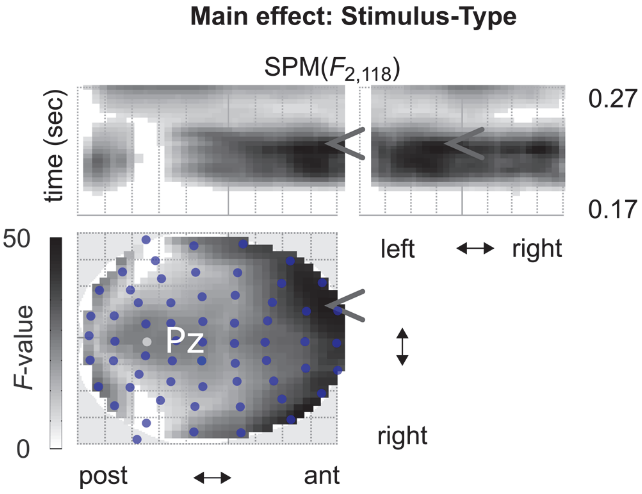

Figure S1-2. Main effect of Stimulus-Type within P2 window (168–272 ms). A paired-sample *t*-test (within-subject) was performed and the results were assessed with F-contrast. The summary statistic scalp-time data volume was thresholded at $p < .001$ (uncorrected) with FWE correction at cluster-level, $p < .05$, based on random field theory. Blue dots mark electrode sites and the grey arrows point to the global maximum. For details, see **Table S1-4**.

Table S1-4. Main effect of factor Stimulus-Type within P2 window (168−272 ms). Statistical results of scalp-time analysis.

Cluster-level		Peak-level				
$P_{FWE\text{-}corr}$	K_E	*F*-value	$P_{uncorrected}$	mm	mm	ms
<.001	10925	49.3	<.001	−17	67	220
.006	736	30.3	<.001	34	−89	208

Statistics: $p_{FWE\text{-}corr}$ = Family-wise-error corrected *p*-values adjusted for search volume. K_E = cluster with *K* elements . Height threshold: $F = 7.33$, $p = .001$ (uncorrected); df = (2, 118); Extent threshold: $k = 103$ bins; Smoothness FWHM = 65.6 88.9+ 51.0 mm mm ms; Expected bins per cluster <*k*> = 135.4; Search vol.: 1976898 = 21635 bins = 10.8 resels; Bin size: 4.2 5.4 4.0 mm mm ms; (resel = 1730 bins).

- **P3b window (320–470 ms)**

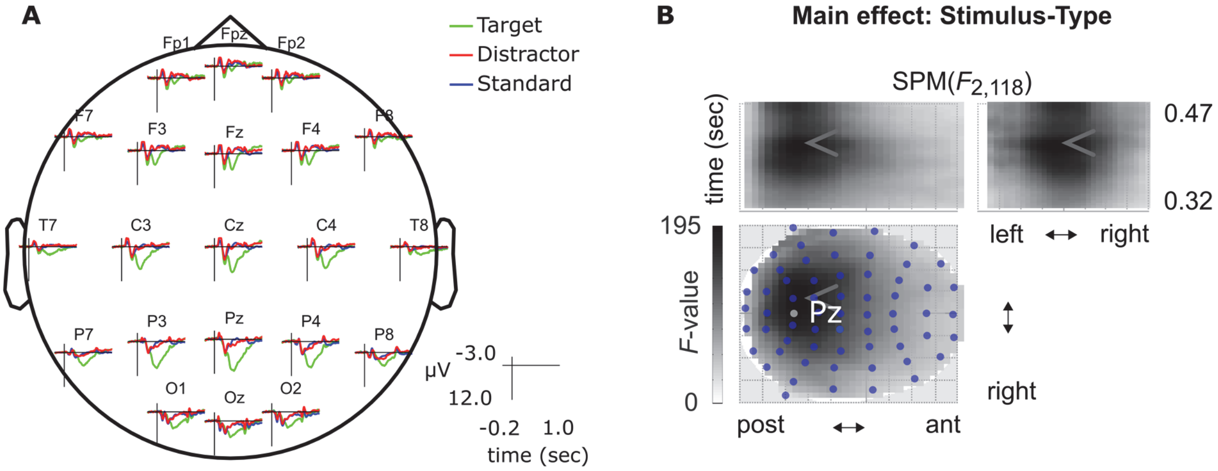

Figure S1-3. (A) Grandaverage waveforms (pooled across subjects and conditions) for target (green), distractor (red), and standard (blue) stimulus type at selected electrode sites (21 out of 64 recorded). Data are referenced to average reference. **(B)** Main effect of Stimulus-Type within P3b window (320–470 ms). A paired-sample *t*-test (within subject) was performed and the results were assessed with F-contrast. The summary statistic scalp-time data volume was thresholded at $p < .001$ (uncorrected) with FWE correction $p < .05$ at cluster-level, based on random field theory. Blue dots mark electrode sites and the grey arrows point to the global maximum. For details, see **Table S1-5**.

Table S1-5. Main effect of factor Stimulus-Type within P3b window (320–470 ms). Statistical results of scalp-time analysis.

Cluster-level		Peak-level				
$P_{FWE-corr}$	K_E	*F*-value	$P_{uncorrected}$	mm	mm	ms
<.001	302040	195.5	<.001	−8	−57	408

Statistics: $p_{FWE-corr}$ = Family-wise-error corrected *p*-values adjusted for search volume. K_E = cluster with *K* elements. Height threshold: $F = 7.33$, $p = .001$ (uncorrected); df = (2, 118); Extent threshold: $k = 0$ bins; Smoothness FWHM = 65.6 88.9+ 51.0 mm mm ms; Expected bins per cluster $<k>$ = 254.5; Search vol.: 2782734 = 30454 bins = 8.2 resels; Bin size: 4.2 5.4 4.0 mm mm ms; (resel = 3251 bins).

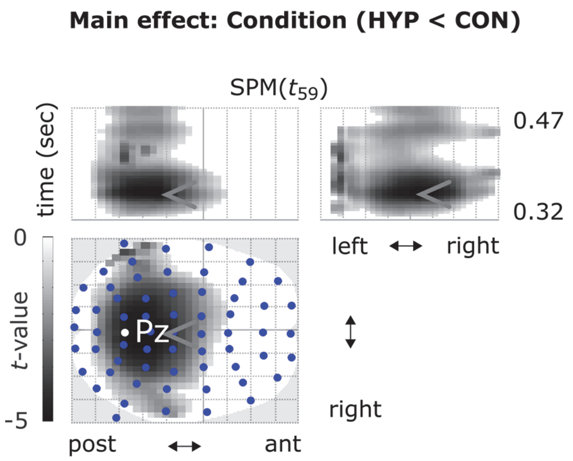

Figure S1-4. Main effect of Condition (HYP < CON) within P3b window (320–470 ms). A one-sample *t*-test was performed and the results were assessed with a *t*-contrast. The summary statistic scalp-time data volume was thresholded at $p < .001$ (uncorrected) with FWE correction at cluster-level, $p < .05$, based on random field theory. Blue dots mark electrode sites and the grey arrows point to the global maximum. For details, see **Table S1-6**.

Table S1-6. Main effect of factor Condition. Statistical results of scalp-time analysis.

Cluster-level		Peak-level				
$p_{FWE\text{-}corr}$	K_E	*t*-value	$p_{uncorrected}$	mm	mm	ms
<.001	8274	4.96	<.001	4	−36	352

Statistics: $p_{FWE\text{-}corr}$ = Family-wise-error corrected *p*-values adjusted for search volume. K_E = cluster with *K* elements. Height threshold: $t = 3.23$, $p = .001$ (uncorrected); df = 59; Extent threshold: $k = 0$ bins; Smoothness FWHM = 71.5 98.7 63.2 mm mm ms; Expected bins per cluster, <k> = 511.1; Search vol.: 2789679 = 30530 bins = 5.5 resels; Bin size: 4.2 5.4 4.0 mm mm ms; (resel = 4880 bins).

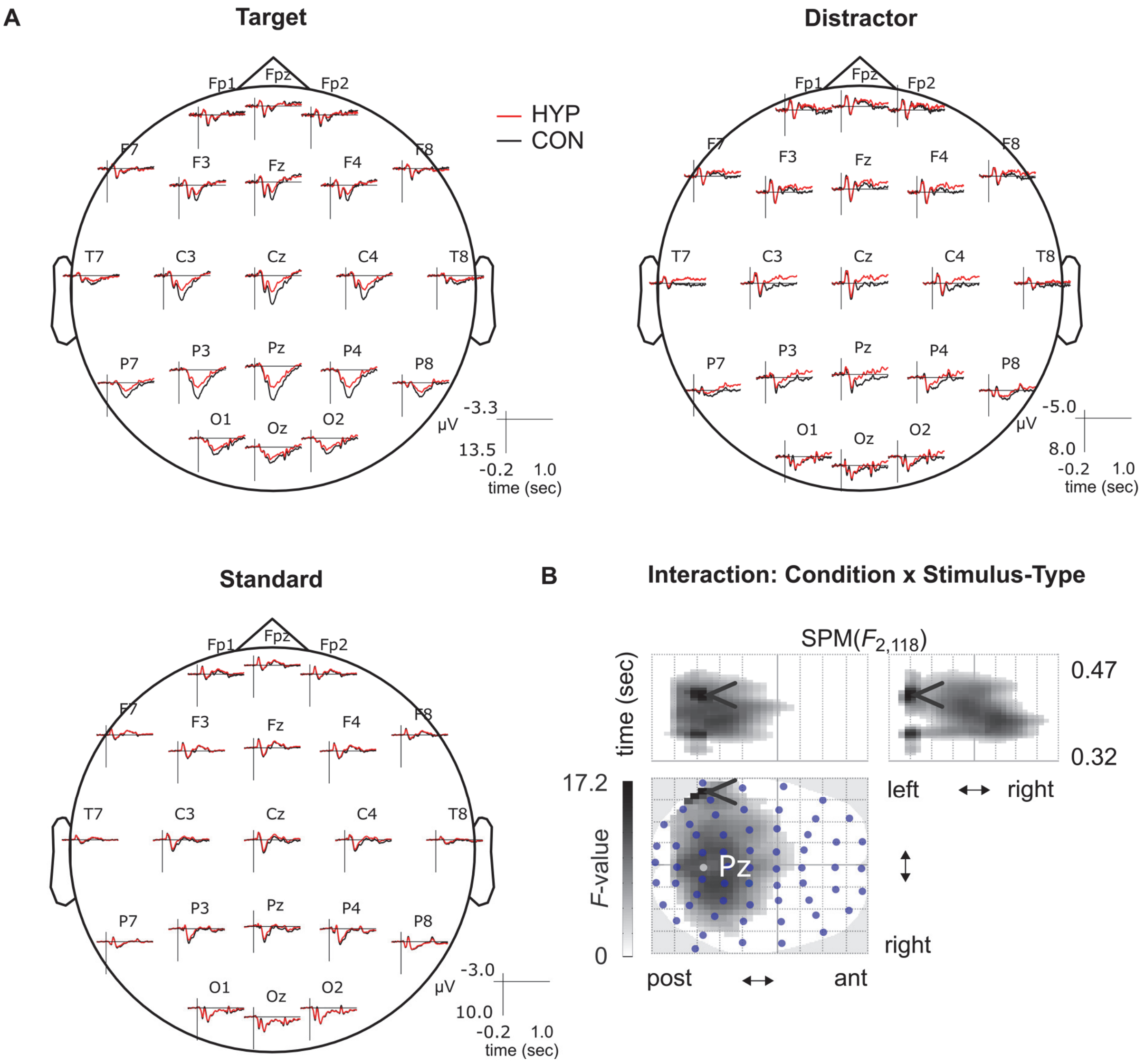

Figure S1-5. (A) Grandaverage waveforms (pooled across subjects and conditions) for target, distractor, and standard stimulus at selected electrode sites (21 out of 64 recorded) in hypnosis (HYP, red) and control (CON, black). Data are referenced to average reference. **(B)** Interaction of Condition x Stimulus-Type within P3b window (320–470 ms). A two-sample *t*-test was performed and the results were assessed with an *F*-contrast. The summary statistic scalp-time data volume was thresholded at $p < .001$ (uncorrected) with FWE correction at cluster-level, $p < .05$, based on random field theory. Blue dots mark electrode sites and the grey arrows point to the global maximum. For details, see **Table S1-7**.

Table S1-7. Interaction of Condition x Stimulus-Type within P3b window (320−470 ms). Statistical results of scalp-time analysis.

Cluster-level		Peak-level				
$P_{\text{FWE-corr}}$	K_E	*F*-value	$P_{\text{uncorrected}}$	mm	mm	ms
<.001	5412	17.2	<.001	−55	−62	408

Statistics: $p_{FWE\text{-}corr}$ = Family-wise-error corrected *p*-values adjusted for search volume. K_E = cluster with *K* elements. Height threshold: $t = 7.33$, $p = .001$ (uncorrected); df = 59; Extent threshold: k =194 bins; Smoothness FWHM = 61.3 87.4 41.2 mm mm ms; Expected bins per cluster, <*k*> = 189.0; Search vol.: 2779810 = 30422 bins = 10.9 resels; Bin size: 4.2 5.4 4.0 mm mm ms; (resel = 2412 bins).

Table S1-8. Statistical results of the t-contrast (HYP < CON) for the scalp-time data separated by stimulus type to disentangle the interaction Condition x Stimulus-Type at sensor-level within the P3b window (320–470 ms).

Stimulus	*t*-Contrasts (HYP < CON)						
	Cluster-level		Peak-level				
	$P_{\text{FWE-corr}}$	K_E	*t-value*	P_{uncorr}	mm	mm	ms
**Target**	<.001	190400	−6.87	<.001	17	−52	372
**Standard**	<.001	731	−3.5	<.001	30	−41	344

Statistics: $p_{FWE\text{-}corr}$ = Family-wise-error corrected *p*-values adjusted for search volume. K_E = cluster with *K* elements; Height threshold: $t = 3.12$, $p = .001$ (uncorrected); df = (1, 295); Extent threshold: $k = 0$ bins; mm = millimeter, ms = millisecond; Smoothness FWHM = 60.4 82.9 46.8 mm mm ms; Expected bins per cluster, <*k*> = 296; Search vol.: 2795161 = 30590 bins = 10.7 resels

SOURCE ANALYSIS

▪ P3b window (320–470 ms)

Table S1-9. Statistical results of the effects of interest (F-contrast) at the source-level within the P3b window (320–470 ms)

	Effects of interest							
	Cluster-level			Peak-level				
						MNI		
Region	p_{Label}	$p_{FWE\text{-}corr}$	K_E	F-value	p_{uncorr}	mm	mm	mm
r OFG	.27	<.001	567	181.6	<.001	28	−84	−7
l SPL	.36	.008	73	117.9	<.001	−38	−40	60
r SPL	.39	.008	73	117.8	<.001	37	−37	59
CC	.19	.030	49	101.7	<.001	1	−25	25
r Hipp	.10	.003	92	86.6	<.001	10	−11	−18
l Hipp	.09	.002	94	85.8	<.001	−11	−13	−19
l LOC	.69	.037	45	81.8	<.001	−31	−68	49
r LOC	.66	.039	44	81.0	<.001	34	−67	46
CC	.15	.021	55	76.0	<.001	0	−38	18
r PCG	.34	.002	97	71.6	<.001	15	−26	67
l PCG	.38	.025	52	71.4	<.001	−14	−27	70
l LV	.58	.021	55	52.8	<.001	0	7	12
l PFC/FP	.26	.002	101	52.5	<.001	−16	55	20
r PFC/FP	.50	.005	83	52.0	<.001	16	58	21
r FLC/FP	.12	.004	85	25.8	<.001	40	39	−3
l FLC/FP	.26	.030	49	18.1	<.001	−44	39	−8

HYP = hypnosis; CON = control; MNI = Montreal Neurological Institute Coordinate System; l = left; r = right; OFG = occipital fusiform gyrus; SPL = superior parietal lobule; CC = posterior cingulate cortex; Hipp = Hippocampus; LOC = lateral occipital cortex; PCG = precentral gyrus; LV = lateral ventricle; PFC = prefrontal cortex, FP = frontal pole; FLC = frontolateral cortex. p_{Label} = probability of the vertex being a member of the different labelled regions within the Harvard-Oxford Subcortical/Cortical Structural Atlas. K_E = cluster with K elements; Statistics: $p_{FWE\text{-}corr}$ = Family-wise-error corrected p-values adjusted for search volume. Height threshold: $F = 5.95$, $p = .001$ (uncorrected); df = [2.7, 293.8]; Extent threshold: k = 40; FWHM = 4.8 4.8 4.8 (vertices); Expected bins per cluster, $<k> = 18.0$; Search vol.: 8196 = 75.7 resels (resel = 108 vertices).

Table S1-10. Statistical results of the *t*-contrast: Target > Standard in CON and HYP at the source-level within the P3b window (320–470 ms)

	CON: Target > Standard							
	Cluster-level			Peak-level				
						MNI		
**Region**	p_{Label}	$p_{FWE\text{-}corr}$	K_E	***t*-value**	p_{uncorr}	**mm**	**mm**	**mm**
r OFG	.42	<.001	470	12.91	<.001	19	−84	−14
l OFG	.42	<.001	430	12.81	<.001	−18	−80	−13
l SPL	.36	.025	61	9.91	<.001	−38	−40	60
r SPL	.39	.025	61	9.91	<.001	37	−37	59
r Hipp	.10	.015	73	8.63	<.001	10	−11	−18
l Hipp	.08	.016	72	8.58	<.001	−11	−13	−19
l PCG	.38	.044	48	8.01	<.001	−14	−27	70
r PCG	.34	.046	47	7.99	<001	15	−26	67
	**HYP: Target > Standard**							
r LG/OFG	.40/.21	<.001	464	10.59	<.001	11	−81	−10
l LG/OFG	.36/.24	<.001	385	10.56	<.001	−13	−77	−13
l Brainstem/Hipp	.09/.08	.015	73	6.95	<.001	−11	−13	−19
r Hipp	.10	.016	72	6.94	<.001	10	−11	−18
l OP	.51	.046	47	6.67	<.001	−25	−95	4
l SPL	.36	.033	55	6.38	<.001	−38	−40	60
r SPL	.39	.033	55	6.37	<.001	37	−37	59

HYP = hypnosis; CON = control; MNI = Montreal Neurological Institute Coordinate System; l = left; r = right; OFG = occipital fusiform gyrus; SPL = superior parietal lobule; Hipp = Hippocampus; PCG = precentral gyrus; LG = lingual gyrus; OP = occipital cortex. p_{Label} = probability of the vertex being a member of the different labelled regions within the Harvard-Oxford Subcortical/Cortical Structural Atlas. K_E = cluster with K_E elements; Statistics: $p_{FWE\text{-}corr}$ = Family-wise-error corrected *p*-values adjusted for search volume. Height threshold: $T = 3.12$, $p = .001$ (uncorrected); df = [1, 293.8]; Extent threshold: 41; Smoothness FWHM = 4.8 4.8 4.8 (vertices); Expected bins per cluster, <*k*> = 23.2; Search vol.: 8196 = 75.7 resels (resel = 108 vertices).

Table S1-11. Statistics of the effect of hypnosis (HYP<CON) for the target stimulus at source-level.

	Peak-level						Target: HYP < CON						
							Cluster		Peak				
	Amplitude (RMS)			Latency (ms)							MNI		
**Region**	**T (Min/Max)**	**S (Min/Max)**	**D (Min/Max)**	**T (Min/Max)**	**S (Min/Max)**	**D (Min/Max)**	$P_{FWE\text{-}corr}$	K_E	t	P_{uncorr}	**mm**	**mm**	**mm**
**HYP**													
r OFG	0.8 (0.3/1.4)	0.4 (0.1/0.8)	0.6 (0.2/1.2)	384 (134/598)	231 (82/534)	273 (54/598)	.020	67	−6.71	<.001	19	−84	−14
l OFG	1.4 (0.5/2.4)	0.7 (0.2/1.3)	1.0 (0.3/2.2)	386 (134/598)	235 (110/534)	278 (54/598)	.015	74	−6.64	<.001	−22	−78	−7
l SPL	1.9 (0.4/4.3)	0.8 (0.1/1.9)	1.4 (0.2/2.6)	434 (102/598)	298 (114/594)	340 (54/586)	.033	55	−6.45	<.001	−38	−40	60
r SPL	1.9 (0.4/4.3)	0.8 (0.1/1.9)	1.4 (0.2/2.6)	434 (102/589)	298 (114/594)	340 (54/586)	.033	55	−6.45	<.001	37	−37	59
**CON**													
r OFG	1.0 (0.3/1.7)	0.4 (0.1/1.0)	0.6 (0.1/1.1)	389 (290/578)	240 (102/330)	296 (98/586)							
l OFG	1.8 (0.6/3.0)	0.7(0.3/1.8)	1.0 (0.2/1.9)	389(290/582)	238 (102/330)	298 (98/586)							
l SPL	2.5 (0.5/5.3)	0.9 (0.2/2.1)	1.6 (0.5/3.0)	438 (138/598)	312 (106/598)	323 (90/590)							
r SPL	2.5 (0.5/5.3)	0.9 (0.2/2.1)	1.6 (0.5/3.0)	438 (138/598)	313 (106/598)	323 (90/590)							

Amplitude and latency values represent mean (Min/Max). HYP = hypnosis; CON = control, l = left; r = right; OFG = occipital fusiform gyrus; SPL = superior parietal lobule. RMS = root mean square (a.u.); T = Target; S = Standard; D = Distractor; MNI = Montreal Neurological Institute Coordinate System; $p_{FWE\text{-}corr}$ = Family-wise-error corrected *p*-values adjusted for search volume. K_E = cluster with *K* elements; Height threshold: $T = -3.12$, $p = .001$ (uncorrected); df = (1, 293.8); Extent threshold: 41; Smoothness FWHM = 4.8 4.8 4.8 (vertices); Expected bins per cluster, <*k*> = 23.2; Search vol.: 8196 = 75.7 resels (resel = 108 vertices)

Relation between source activity and counting accuracy

To assess the relation between source activities of the P3b component and behavioural performance, we calculated difference scores (HYP−CON) for source activations within different brain regions contributing to the target P3b component and for counting accuracy. The correlations between difference scores in counting accuracy and source activities of different brain areas are listed in Table S1-10 and illustrated in Figure S1-6.

Table S1-12. Correlation between source activity differences and counting accuracy differences between the HYP and CON condition for different brain regions contributing to the target P3b component.

			MNI		
**Region**	***r***	***p***	**mm**	**mm**	**mm**
l OFG	**−0.49**	<.001	−18	−80	−13
r OFG	**−0.50**	<.001	19	−84	−14
l SPL	**−0.27**	0.03	−38	−40	60
r SPL	**−0.27**	0.03	37	−37	59
l Hipp	**−0.48**	<.001	−11	−13	−19
r Hipp	**−0.48**	<.001	10	−11	−18
l PCG	−0.20	0.12	−14	−27	70
r PCG	−0.20	0.12	15	−26	67
l PFC	−0.11	0.58	−16	55	20
r PFC	−0.11	0.58	16	58	21

l = left; r = right; OFG = occipital fusiform gyrus; SPL = superior parietal lobule; Hipp = Hippocampus; PCG = precentral gyrus; PFC = prefrontal cortex

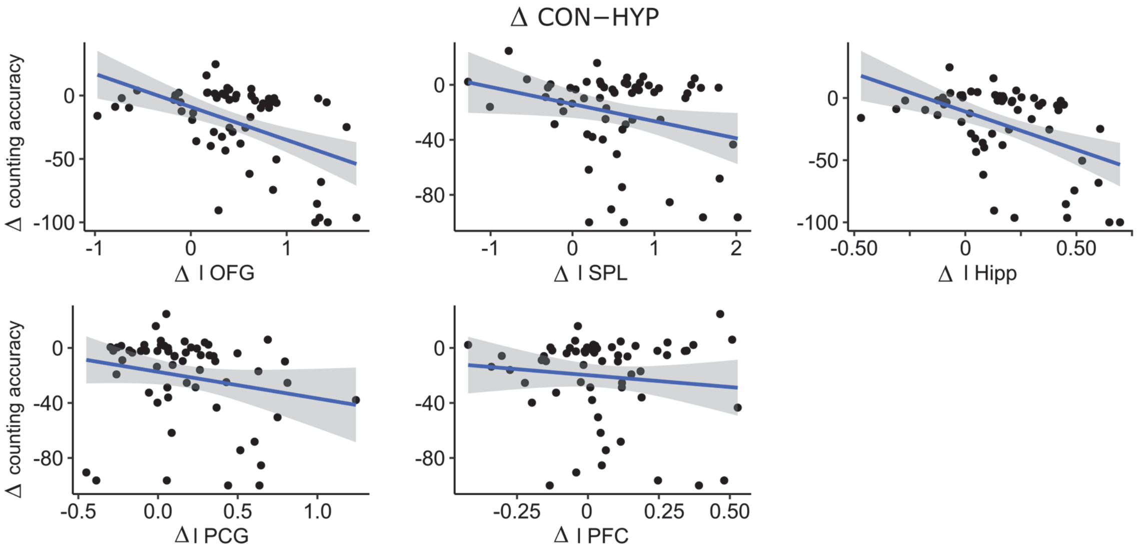

Figure S1-6. Correlation between difference scores Δ of source activity and counting accuracy between the CON and HYP condition of the left hemisphere. l = left; OFG = occipital fusiform gyrus; SPL = superior parietal lobule; Hipp = Hippocampus; PCG = precentral gyrus

CONNECTIVITY ANALYSIS: P3b

MVAR model estimation and validation. Figure S1-7 depicts the frequency distribution of the optimal model order separately for HYP and CON. The average model order across all subjects was $M_p \approx 13$ (*SD*=1.53) for HYP, and $M_p \approx 13$ (*SD*=1.45) for CON condition. Since model order was separately determined for the hypnosis and control condition in each subject, the larger order of both conditions was selected for the MVAR model estimation of each subject so that both conditions were matched in terms of model order ($M_p \approx 14$, Min: 10, Max: 20).

The results of the MVAR model validation for HYP and CON averaged across all subjects are summarized in Fig S1-7B. On average, ≈76% of the models in HYP and ≈81% in CON passed the residual-whiteness tests within the latency windows of interest (200–600 ms) indicating that the MVAR models were capturing the temporal dynamics in a large percentage of subjects during the respective latency windows. However, there was no subject in which all the models passed the residual-whiteness tests across the 86 sliding windows of the entire epoch (−1.0 to 1.0 s). The percent consistency, indicating the similarity between the correlational structure of the real data and the modeled data, was uniformly around 90% across all windows for both HYP and CON indicating a high degree of similarity. In all subject's models, the stability index was negative indicating that the models were stable across the entire epoch.

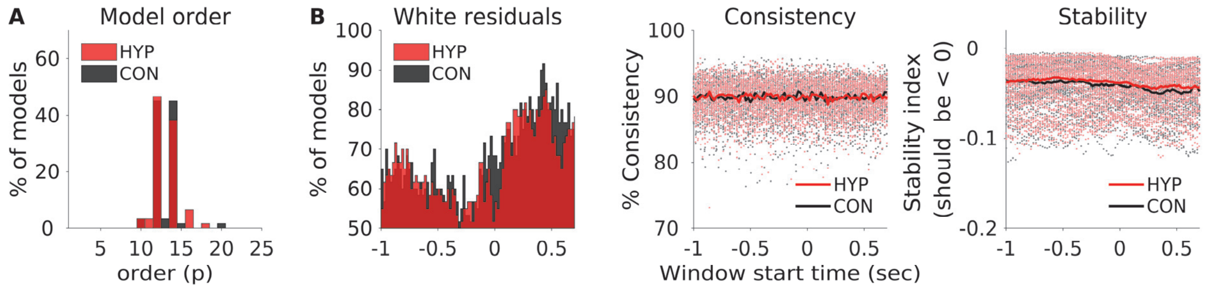

Figure S1-7. Model estimation and validation criteria for the hypnosis (HYP, red) and control (CON, black) condition averaged across all subjects ($n = 60$). **(A)** Frequency distribution (in %) of optimal model order across all subjects for HYP and CON. **(B)** Left panel: The whiteness of the MVAR model residuals was tested with the autocorrelation function (ACF) across the time windows (86 in total) of the epoch. Within the latency of interest (200 to 600ms) a large percentage of MVAR models passed the residual-whiteness tests in HYP (≈76%) and CON (≈81%) indicating that the temporal dynamics were captured by the models within these time windows. Middle panel: The percent consistency as a function of time. The consistency indicates the amount of the correlational structure in the data captured by the model. Both in HYP and CON the measure was reliably around 90% indicating a high degree of consistency. Right panel: The Stability index as a function of time. The index is defined as the logarithm of the largest eigenvalue (λ of the AR model. A negative quantity means that the largest eigenvalue satisfies the condition $|\lambda| < 1$ which implies that, the model is stable. In all subjects, the MVAR models were stable across the entire epoch.

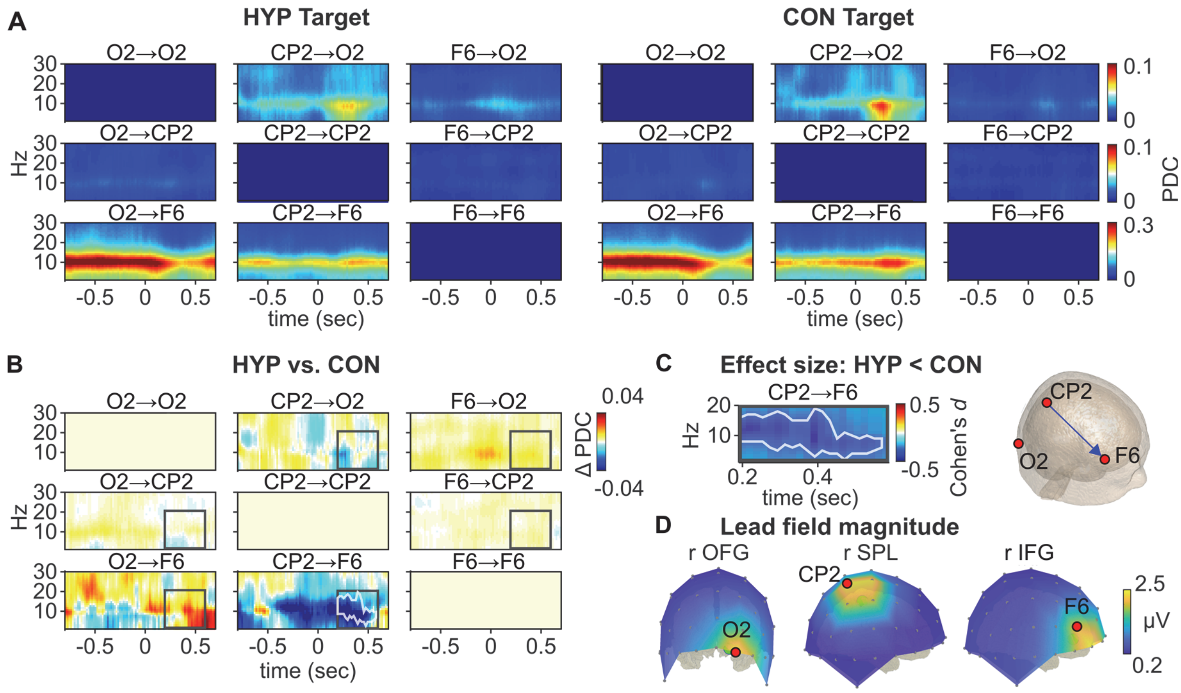

Figure S1-8. Spectro-temporal connectivity within the network of three electrodes (O2, CP2, F6) at the right hemisphere across all subjects (*n* = 60). **(A)** Evolution of the pairwise connectivity during the hypnosis (HYP, left) and control (CON, right) condition for the target stimulus. The connectivity values represent the time-variant normalized partial directed coherence (PDC) from 1 to 30 Hz within the latency range of −800 to 700 ms relative to the target stimulus. **(B)** Absolute difference of the connectivity matrices between HYP and CON. Warmer colors (red) indicate HYP > CON and cooler colors (blue) CON > HYP. The grey rectangle marks the spectro-temporal region of interest (ROI) that was used for statistical comparison of both conditions. **(C)** Effect size expressed as Cohen's *d* for the statistically significant ROI difference ((cluster statistic $\Sigma_t = -504.33$, $p = .017$) in spectrotemporal connectivity between HYP vs. CON. White lines in (B) and (C) encircle the significant spectro-temporal region at $p < 0.05$ corrected for multiple comparisons in the time-frequency plane using cluster-based statistics. **(D)** Lead field magnitude (μV) of three source cluster peaks located in the right hemisphere: the occipital fusiform gyrus (r OFG), the superior parietal lobe (r SPL), and the inferior frontal gyrus (r IFG). The maximum lead field magnitude of these source cluster peaks defined three electrodes that were selected for the connectivity analysis.

REFERENCES

1. Friston K, Harrison L, Daunizeau J, Kiebel S, Phillips C, Trujillo-Barreto N, et al. Multiple sparse priors for the M/EEG inverse problem. Neuroimage. 2008;39(3):1104-20.

2. Lopez JD, Litvak V, Espinosa JJ, Friston K, Barnes GR. Algorithmic procedures for Bayesian MEG/EEG source reconstruction in SPM. Neuroimage. 2014;84:476-87.

3. Scherg M, Ebersole JS. Brain source imaging of focal and multifocal epileptiform EEG activity. Neurophysiol Clin. 1994;24(1):51-60.

4. Miltner W, Braun C, Johnson R, Jr., Simpson GV, Ruchkin DS. A test of brain electrical source analysis (BESA): a simulation study. Electroencephalogr Clin Neurophysiol. 1994;91(4):295-310.

5. Dale AM, Sereno MI. Improved localizadon of cortical activity by combining eeg and meg with mri cortical surface reconstruction: A linear approach. J Cognitive Neuroscience. 1993;5(2):162-76.

6. Belardinelli P, Ortiz E, Barnes G, Noppeney U, Preissl H. Source reconstruction accuracy of MEG and EEG Bayesian inversion approaches. PLoS One. 2012;7(12):e51985.

7. Henson RN, Wakeman DG, Litvak V, Friston KJ. A Parametric Empirical Bayesian Framework for the EEG/MEG Inverse Problem: Generative Models for Multi-Subject and Multi-Modal Integration. Front Hum Neurosci. 2011;5:76.

8. Phillips C, Mattout J, Rugg MD, Maquet P, Friston KJ. An empirical Bayesian solution to the source reconstruction problem in EEG. Neuroimage. 2005;24(4):997-1011.

9. Friston KJ, Mattout J, Trujillo-Barreto N, Ashburner J, Penny W. Variational free energy and the Laplace approximation. Neuroimage. 2007;34(1):220-34.

10. Lopez JD, Barnes GR, Espinosa JJ. Single Meg/Eeg Source Reconstruction with Multiple Sparse Priors and Variable Patches. Dyna-Colombia. 2012;79(174):136-44.

11. SPM. Group Analysis 2018 [Available from: https://en.wikibooks.org/w/index.php?title=SPM/Group_Analysis&oldid=3373190.

12. Delorme A, Mullen T, Kothe C, Acar ZA, Bigdely-Shamlo N, Vankov A, et al. EEGLAB, SIFT, NFT, BCILAB, and ERICA: New Tools for Advanced EEG Processing. Comput Intel Neurosc. 2011.

13. Ding M, Bressler SL, Yang W, Liang H. Short-window spectral analysis of cortical event-related potentials by adaptive multivariate autoregressive modeling: data preprocessing, model validation, and variability assessment. Biol Cybern. 2000;83(1):35-45.
