# Supplementary material for: Suggested visual blockade during hypnosis: Top-down modulation of stimulus processing in a visual oddball task
Source: PLoS One. 2021 Sep 15;16(9):e0257380. doi: 10.1371/journal.pone.0257380 (PMC8443036; doi:10.1371/journal.pone.0257380)
Supplement: S2 File — (PDF) [file pone.0257380.s002.pdf]

Supplemental information I to the article:

Suggested visual blockade during hypnosis:
Top-down modulation of stimulus processing in a visual oddball task

Marcel Franz[1*], Barbara Schmidt[1], Holger Hecht[1], Ewald Naumann[2] and Wolfgang H.R. Miltner[1]

[1] Institute of Psychology, Friedrich Schiller University of Jena, Jena, Germany

[2] Institute of Psychology, University of Trier, Trier, Germany

English translation of the hypnosis induction and suggestions used in this experiment

Induction of hypnotic state (trance)

„Can you hear me well through the headphones? (Waiting for a response)
Then let's start with our hypnosis session. I want you to relax as much as possible and sit comfortably in the chair for the next time. You already know a lot of what you will experience from your previous hypnosis session where we tested your suggestibility. The session today is, so to speak, a continuation of the last session with the aim of examining more closely how you and your brain will react to different hypnotic suggestions. Of course, nothing will happen here that is not in line with your own will or that could compromise you in any way. Just pay attention to what happens and try to allow the experiences that are suggested to you. Don't push yourself to anything that doesn't work, but don't try to hold back things that work. You can't go wrong. Just be completely relaxed.

Do you see the small lens above the glass window in front of you? In the following, I will call this lens "sign". I would ask you to relax in the chair now, and to look at the sign, and listen to my voice. I will give you some instructions that will help you to relax and gradually get into a hypnotic state.
Please keep your eyes on the sign and keep paying attention to my words as you look at the sign. You can only get into a hypnotic state if you want to. Try your best to focus on the sign and pay close attention to my words. If your thoughts wander, bring your focus back to the sign and to my words. Notice how the sign changes, how it may sometimes become blurred, sometimes clearer. Whatever goes through your head, allow it, but keep fixing the sign.

Relaxing in hypnosis is very similar to the state just before falling asleep. But you will not sleep in the usual sense because you will continue to hear my voice and you will be able to focus your attention on what I am telling you.

You are pleasantly relaxed, but you will relax much more, much more. Just pay close attention to my voice. Sometimes, my voice may seem to change, or it may sound like it's far away. That's fine. Don't worry. If you start feeling sleepy, that's fine too. Accept whatever happens and just keep listening to my voice as you relax more and more.

As you imagine relaxation, your muscles will relax. Start with the right foot, relax it and then go on to the muscles of your right leg and relay them ... now the muscles of your left leg ... relax completely. Relax your right hand … your forearm … your upper arm … and your shoulder ... Now your left hand ... and your forearm ... and your upper arm ... and shoulder ... Relax your neck … and your chest ... completely relaxed.

As you relax more and more, your body will feel heavy or maybe numb. You will start to feel this numbness or heaviness in your legs and feet ... in your hands and arms ... throughout your body ... and you might get the impression as of sinking deeper into your chair. The chair is stable, it will hold your body while it feels heavier and heavier.

Your eyelids will start feeling heavy … heavy and tired. You start to feel tired … and sleepy. Your eyes might burn a little … and your eyelids feel very heavy. Your eyes are blurred from the effort. You can hardly see the sign anymore; your eyes are so strained. Soon you won't be able to keep your eyes open. Your eyelids are heavy. Become heavier and heavier. They push down, deeper and deeper. There seem to be weights on the eyelids that push them deeper and deeper…. Your eyes flicker ... flicker ... and finally close, close … Your eyes are now closed. Just keep your eyes closed until I ask you to open them again.

You are relaxed, very relaxed. You can relax even more, if you just let yourself go. You can reach a state of even deeper, complete relaxation. You become increasingly tired and sleepy. And you feel a pleasant numbness and heaviness in your entire body. You start to feel so relaxed, so sleepy. It's easier to avert your thoughts from other things and solely focus your attention onto my voice. Soon, you will only sleepily listen to my voice as you relax more and more.

Now I want you to pay attention to your breathing ... Take a deep breath ... deep breath. Then hold your breath ... And then exhale through your mouth. Maybe you can already feel how calmness and relaxation spread in your body ... Now breathe in and out calmly and evenly - in and out ... When you breathe in, your abdominal wall bulges upwards, and when you breathe out, it falls back easily down ... on ... and off ... on ... and off. Feel, how this inhalation and exhalation goes hand in hand with rest and relaxation! If you continue to breathe calmly like this, sooner or later you will find yourself in a very

pleasant inner peace without having to do anything for it ... You breathe in and out ... in and out. Whenever you inhale, you take in oxygen and energy ... And whenever you exhale, you relax more deeply and can let go ... With every breath you can let go more ... with every single breath ... You exhale and let go of everything that disturbs you ... you exhale and inner peace becomes more and more - come of its own accord ... and with each breath, the relaxation becomes deeper and deeper ... as deep as it is now is possible.
You don't have to do anything other than pay attention to your breathing and feel how your breath flows in and out ... in and out ... And you can imagine how your breathing air flows through your nose through your windpipe to your lungs and back again...
Some focus their attention on the muscles of the body and feel how they relax more and more deeply with each breath, as if breathing tells the muscles to relax even further ... You don't need to do anything at all ... just feel and remember. Often you can feel the relaxation first in the temple area. It moves over the forehead to the eyes ... to the root of the nose over the cheeks and the nostrils ... over the lips into your mouth.

And this relaxation also extends to the neck, because with every breath, the muscles of your neck become more relaxed ... You don't have to do anything. Just feel and notice how the relaxation flows - like a current - over your shoulders, down your back ... Every time you exhale you can feel the relaxation - in the upper arms ... in your forearms ... and finally joins the fingers, the palm of your hand ... right into your fingertips ... And when you exhale, the air flows gently and smoothly back out through your nose … With every breath, rest and relaxation become more and more ... If you observe your nose, you can notice how the air breathes in and out through your nose ... And you can feel how gradually the whole head area, the upper body and your arms and hands are gripped by a deep feeling of relaxation and calmness ... When you breathe in, you can have the feeling of becoming lighter ... This is quite normal ... You may have noticed that to exhale takes a little longer than inhaling ... So you sink deeper ... and deeper ... and deeper with each breath.

You are relaxed, very relaxed. Your whole body feels heavy and relaxed. You realize a pleasantly warm feeling all over your body as you become more and more tired and sleepy. Sleepy. Tired. Tired and sleepy. Keep your thoughts on what I'm saying; listen to my voice. Soon you will think of nothing but my voice and the words I say as you relax more and more. There is nothing you need to worry about now. Nothing but what my voice tells you seems important, nothing else is important now. Even my voice may sound a little strange, as if it came from a dream, as you sink deeper into this numbness, this heaviness of deep relaxation. Relax completely ...

I will now start counting from 1 to 10 soon. As I count, you will notice yourself falling deeper and deeper into a restful sleep. But you will still be able to do all the things, I ask you to do without waking

up ... 1… you start to relax even more deeply… 2…. Deep, deep into a deep healthy sleep ... 3, 4 ... sleeping deeper and deeper ... 5, 6, 7, ... You sink into a deep, deep sleep. Nothing will bother you ... Please direct your thoughts to my voice and to the things I tell you. You will experience many of the things I describe to you ... 8, 9, 10 ... deep asleep. You won't wake up until I ask you to.

Now, I want you to realize that if I ask you to do so, you will be able to speak, move, and even open your eyes while remaining in the hypnotic state you are in now. Whatever you do, you will stay hypnotic until I tell you otherwise ...

Test of Trance

Now please extend your right arm at shoulder height, palm up. Just like that. Please focus on your right hand, how it feels, what is going on in it. Notice whether it feels a little numb or not, or maybe tingles. Now please imagine that you have something heavy in your hand, maybe a heavy ball ... something heavy. Shape your fingers around it as if you were holding this heavy object you imagine.
Now the hand and arm feel heavy as if a weight is pushing them down ... the hand and arm are starting to move down ... as if they are being pushed down ... they are sinking ... sinking ... lower and lower ... The arm is becoming tired and strained ... deeper ... slowly but surely ... down, down ... the weight is so big, the hand is so heavy ... you feel the weight more and more ... the arm is too heavy to hold it back ... it moves down, down ... deeper and deeper ...
Good. Simply return your hand to its original resting position and relax. Whatever you do, you will stay hypnotic until I tell you otherwise ...

<u>Sequence 1: first hypnosis, then baseline</u>
3 figures will appear on the screen in quick succession: red circles, yellow triangles and blue squares. I would like to ask you to try to count the blue squares, nothing else. Just count the blue squares. Then, after a short pause, red circles and blue squares are shown again in quick succession. You should just look at them, nothing else. Just look at it. Before that, however, I will give you further hypnotic suggestions. Just pay attention to what happens and try to allow the experiences that I suggest to you right away. Don't push yourself to anything that doesn't work, but don't try to hold back things that work.
<u>Sequence 2: first baseline, then hypnosis</u>
The characters you already know from before will appear on the screen one after the other: circles, triangles and squares. But this time in different colors, namely the circles are purple, the triangles are brown and the squares are green. Can you still remember your previous task? You should count the squares. I want to ask you to try that again, just like before. So just count the - this time green - squares. And afterwards, after a short break, circles and squares will reappear in quick succession. Just like before, you should just look at them, nothing else. Before that, however, I will give you further hypnotic suggestions. Just pay attention to what is happening and try to allow the experiences that I suggest to

you right away. Don't push yourself to anything that doesn't work, but don't try to hold back things that work.

Please try opening your eyes very slowly. Very slowly ... Good. Now please look at the middle of the screen. Do you see the hexagon in front of you? I will refer to it as a "symbol" below.

Perceptual blockade suggestion

I want to try something with you now. Let's see if you can ignore the symbol on the screen with your eyes open. (A six-sided figure is presented on the screen in front of the participant). This might sound strange as you are looking directly at the symbol on the screen. But when you are hypnotized, you can tell your brain to block things in front of your eyes so that you can't see them anymore. Maybe you know from your daily routine that you can sometimes totally fade out things when you concentrate strongly on something else. It will be harder and harder to see the symbol. Sooner or later you will be able to fade out the symbol. Just be curious. To make that easier for you, you could imagine that I put a wooden board between your eyes and the screen. The wooden board will block your vision and prevent you from seeing the screen. Just try to imagine that this wooden board moves slowly in front of your eyes so that the screen completely disappears behind it. The board is moving slowly in front of your eyes now.

You see the board in front of you, its texture and its color. Look closely! What kind of wood is it? Is it dark or light? Smooth or rough? Is there anything carved into it? The wooden board gets more and more real and definite in front of your eyes. You notice how hard it gets to see on the screen. You realize that you can just tell yourself to ignore the symbol. It gets harder and harder to see it as if it hides from you. It hides behind the wooden board which you see clearer and clearer now. The screen faints more and more. You notice that it gets harder and harder to see the screen.

I will start to count from one to ten and then you will see nothing on the screen anymore. One - it gets harder and harder to see the screen - two - three - the wooden board is so clear now that you can hardly see behind it - four - five - six - you are unable to look behind the board, it blocks your vision - seven - eight - nine - ten

Now you don’t see the screen anymore, the wooden board completely blocks your vision. You wonder how you made it to ignore everything else, that you are unable to see the symbol. The only thing you see is the wooden board. How fascinating it is that you can block your vision with the power of your mind! Keep on looking at the board. Look closely. You won’t see behind it until I tell you something differently. Just relax.

(Now the visual oddball paradigm starts)

You realize that there might be something behind the wooden board, but you don’t really care. You don’t have to care about it. Just relax. It is easy for you to ignore everything but the wooden board.

Afterwards: It is still very difficult for you to look at the screen. As if he eludes your eyes, hides from you ... But that's fine, you don't need to worry ... just stay relaxed ...

Steady State Stimulus Condition

Very good, now this part is done!

Cancellation of trance

I would like to ask you to slowly slide the board away from your eyes ... the board keeps disappearing from your field of vision and you start to see the screen more clearly again ... your view becomes ever clearer ... the screen is now again clearly and clearly visible to you.

Please stay relaxed and listen to me. Enjoy these moments a little ... just relax ...

I will start counting downwards from 10 to 1 ... you will feel how your relaxation turns into a pleasant feeling of being rested and how you end up feeling fresh and alert.
Well, I'm starting to count: 10 ... 9 ... 8 ... 7 ... 6 ... 5 ... 4 ... 3 ... 2 1 … Now take a deep breath. A feeling of freshness in the lungs accompanies your breathing and you feel increasingly relaxed. I want you to consciously take a few more breaths and breathe deeply through your nose and exhale audibly through your mouth. When you are on the penultimate of these breaths, you can tighten your muscles and loll yourself and you are back in the here and now.
→ How many blue (1) or green (2) squares did you see?

Sequence 1: first hypnosis, then baseline

So now comes another part of the experiment where you should count all the green squares.
After a short pause, circles and squares will reappear in quick succession. Just like before, you should just look at them, nothing else.
